# Deficiency of SIAH1 promotes the formation of filopodia by increasing the accumulation of FASN in liver cancer

**DOI:** 10.1038/s41419-024-06929-7

**Published:** 2024-07-29

**Authors:** Zhiyi Liu, Qinghe Hu, Kuan Cao, Jun Sun, Licheng Cui, Mengxuan Ji, Wengang Shan, Weichao Yang, Guowei Zhang, Zilu Tian, Hengliang Shi, Bin Zhang, Renhao Wang

**Affiliations:** 1 Institute of Digestive Diseases, Xuzhou Medical University, Xuzhou, Jiangsu China; 2 Research Center of Digestive Diseases, The Affiliated Hospital of Xuzhou Medical University, Xuzhou, Jiangsu China; 3 Department of General Surgery, The Affiliated Hospital of Xuzhou Medical University, Xuzhou, Jiangsu China; 4 Hepatobiliary Surgery, Department of General Surgery, Huashan Hospital & Cancer Metastasis Institute, Fudan University, Shanghai, China; 5 Central Laboratory, The Affiliated Hospital of Xuzhou Medical University, Xuzhou, Jiangsu China

**Keywords:** Ubiquitylation, Deubiquitylating enzymes

## Abstract

It has been shown that the formation of filopodia is a key step in tumor cell metastasis, but there is limited research regarding its mechanism. In this study, we demonstrated that fatty acid synthase (FASN) promoted filopodia formation in liver cancer cells by regulating fascin actin-bundling protein 1 (FSCN1), a marker protein for filopodia. Mechanistically, on the one hand, the accumulation of FASN is caused by the enhanced deubiquitination of FASN mediated by UCHL5 (ubiquitin c-terminal hydrolase L5). In this pathway, low expression of SIAH1 (Seven in absentia homolog 1) can decrease the ubiquitination and degradation of ADRM1 (adhesion regulating molecule 1) thereby increasing its protein level, which will recruit and activate the deubiquitination enzyme UCHL5, leading to FASN undergo deubiquitination and escape from proteasomal degradation. On the other hand, the accumulation of FASN is related to its weakened ubiquitination, where SIAH1 directly acts as a ubiquitin ligase toward FASN, and low expression of SIAH1 reduces the ubiquitination and degradation of FASN. Both the two pathways are involved in the regulation of FASN in liver cancer. Our results reveal a novel mechanism for FASN accumulation due to the low expression of SIAH1 in human liver cancer and suggest an important role of FASN in filopodia formation in liver cancer cells.

## Introduction

Liver cancer is a common type of malignant tumor of the digestive system. It has been reported that the 5-year overall survival rate of patients in China is only 12.5% [1, 2], indicating a poor prognosis largely because of recurrence and metastasis. Therefore, it is necessary to explore the molecular mechanism of liver cancer metastasis and identify more effective molecular targets.

Cell migration, which is the self-directed movement of cells using their pseudopodia, is necessary for tumor invasion and metastatic growth [3]. Studies have shown that the formation of filopodia plays an important role in the migration of liver cancer cells [4, 5]. Fatty acid synthase (FASN), a key enzyme required for the synthesis of fatty acids and some biologically important lipid precursors, can regulate metabolism, cell survival and proliferation, DNA replication, transcription, and protein degradation [6–8]. It has been shown that FASN can promote the proliferation, metastasis, and apoptosis of liver cancer cells, therefore playing a key role in liver cancer progression [9–11]. Recent studies revealed that FASN can directly regulate FSCN1 (fascin actin-bundling protein 1), a protein related to the formation of filamentous pseudopodia, lamellar pseudopodia, and microspines and coding of cytoskeletal proteins, thereby promoting the migration and invasion of liver cancer cells [12, 13]. However, the effect of FASN on filopodia formation and the regulatory mechanism of FASN require further exploration.

Ubiquitination refers to the process by which ubiquitin classifies proteins in cells relying on the action of a series of special enzymes, including ubiquitin-activating enzymes (E1), ubiquitin-binding enzymes (E2), ubiquitin-ligases (E3), and deubiquitinating enzymes (DUBs) and, subsequently, selects and modifies the target proteins specifically. Substrates modified by E3s are either degraded by the ubiquitin–proteasome system (UPS), as in most cases, or are altered in their interactions, localization, or enzyme activity. It has been shown that 80–90% of intracellular proteins are degraded by the UPS pathway—a post-translational modification in eukaryotes that regulates important biological processes such as cell cycle, metabolism, proliferation, apoptosis, signal transduction, and DNA damage repair [14, 15]. Although the regulation of FASN by UPS has been reported, research is still in its nascent stage, and further studies are required.

In this study, we highlight the effect and mechanism of FASN on filopodia formation in liver cancer cells. We have revealed the molecular mechanism by which loss of SIAH1 in liver cancer regulates the protein stability of FASN through two pathways: promoting deubiquitination and reducing ubiquitination. Collectively, our findings suggest that the SIAH1-FASN–FSCN1 axis is crucial for filopodia formation in liver cancer cells, and this discovery provides a promising strategy for the treatment of liver cancer.

## Results

### FASN promotes filopodia formation in liver cancer cells by regulating FSCN1

**Fig. 1:**
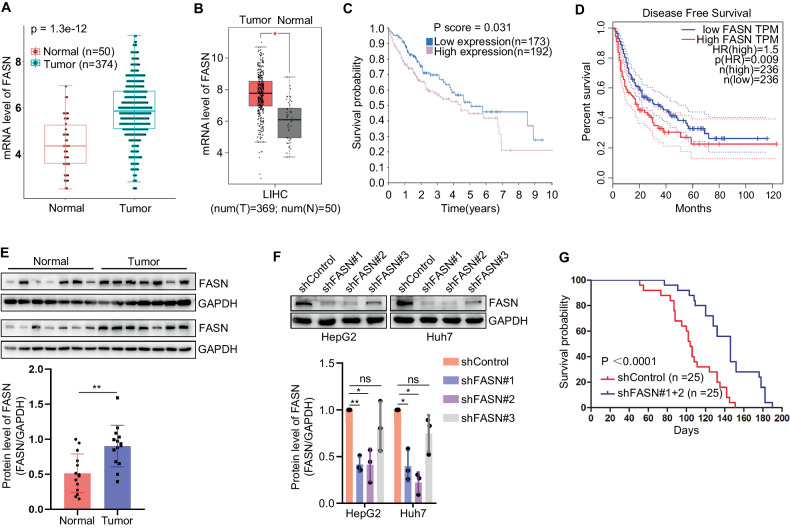
FASN is upregulated in liver cancer and associated with poor prognosis. **A**, **B** FASN mRNA levels in human liver tumor and normal liver tissues in TCGA (T, *n* = 374; N, *n* = 50) and GEPIA (T, *n* = 369; N, *n* = 50) databases. **C** Correlation between FASN mRNA level and human overall survival in TCGA database (high, *n* = 192; low, *n* = 173). **D** Correlation between FASN mRNA level and human disease-free survival in GEPIA database (high, *n* = 236; low, *n* = 236). **E** Protein levels of FASN in human liver tumor (*n* = 14) and normal liver (*n* = 14) tissues. The protein expression levels of target genes were normalized to those of GAPDH (loading control). **F** Representative blots and quantification of FASN expression in human liver cancer cells. **G** Correlation between FASN expression level and mice overall survival (shControl, *n* = 25; shFASN, *n* = 25). *P < 0.05, **P < 0.01.

Multiple studies reported that FASN was upregulated in liver cancer, and associated with the malignant progression and poor prognosis [9–11]. To detect the potential role of FASN in liver cancer, we analyzed the expression level of FASN in human liver cancer tissues and non-tumor tissues using the databases. The mRNA level of FASN in liver cancer tissues was upregulated (Fig. 1A, B). Furthermore, we analyzed the correlation between FASN mRNA level and patient survival. It was found that a high FASN mRNA level was associated with poor prognosis in liver cancer patients (Fig. 1C, D). Subsequently, the clinical tissue samples were extracted to detect whether the protein level of FASN was consistent with mRNA expression. It was found that the protein level of FASN in liver cancer samples was upregulated correspondingly (Fig. 1E). In addition, we constructed a survival model using nude mice and found that low levels of FASN were associated with longer survival time (Fig. 1F, G). Further analysis of clinical data revealed a correlation between the expression of FASN and the clinicopathological characteristics of liver cancer patients. We found that high levels of FASN were associated with tumor size (*P* < 0.05), venous invasion (*P* < 0.05), and direct liver invasion (*P* < 0.05). There was no significant correlation between FASN expression and the remaining pathological features (Table 1).

**Table 1:** Clinicopathological correlation of FASN expression in Human liver cancer.

Clinicopathological features		FASN low	FASN high	*P* value
Gender	Male	28	24	>0.9999
	Female	10	8	
Tumor size, cm	<5	22	10	0.0321*
	≥5	16	22	
Number of tumor nodules	1	30	23	0.5804
	≥2	8	9	
Venous invasion	Present	13	21	0.0158*
	Absent	25	11	
Tumor microsatellite formation	Present	17	18	0.472
	Absent	21	14	
Direct liver invasion	Present	15	22	0.0145*
	Absent	23	10	
Tumor encapsulation	Present	20	14	0.4823
	Absent	18	18	
Cellular differentiation according to Edmondson’s grading	Grades l	16	13	0.9388
	Grades Il–IV	23	18	
Background liver diseases	Normal and chronic hepatitis	17	12	0.7134
	Cirrhosis	13	14	
	Steatohepatitis	8	6	
T stage	T1	11	7	0.5
	T2-4	27	25	
N stage	N0	37	31	＞0.999
	N1	1	1	
M stage	M0	38	32	＞0.999
	M1	0	0	
MVI	M0	23	19	＞0.999
	M1,M2	15	13	

^*^P <0.05. FASN high or low were grouped mainly according to the median protein level.

**Fig. 2:**
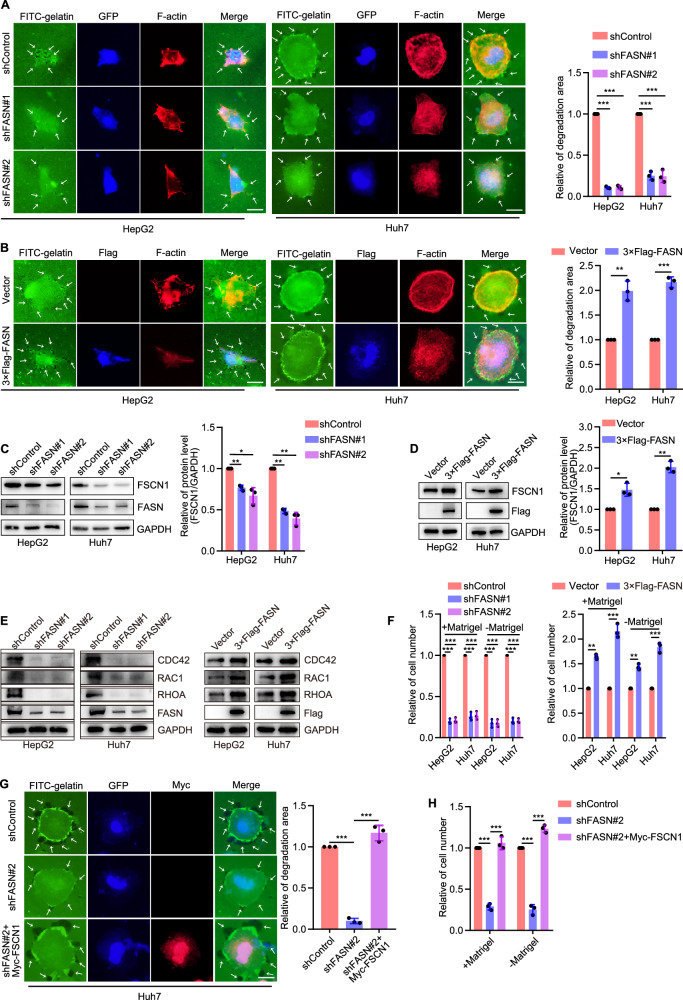
FASN promotes filopodia formation in liver cancer cells by regulating FSCN1. **A** Representative images and quantification of filopodia localization assay in HepG2 and Huh7 cells silencing FASN. Scale bar, 12.5 µm. **B** Representative images and quantification of filopodia localization assay in HepG2 and Huh7 cells overexpressing FASN. Scale bar, 12.5 µm. **C**, **D** Representative blots and quantification of FSCN1 expression in HepG2 and Huh7 cells silencing or overexpressing FASN. **E** Representative blots of CDC42, RAC1 and RHOA expression in HepG2 and Huh7 cells silencing or overexpressing FASN. **F** Quantification of cell invasion and migration while silencing or overexpressing FASN. **G** Representative images and quantification of filopodia localization assay of shControl, shFASN#2, and shFASN#2+Myc-FSCN1 groups in Huh7 cells. Scale bar, 12.5 µm. **H** Quantification of Huh7 cell invasion and migration. *P < 0.05, **P < 0.01, ***P < 0.001.

The high degree of malignancy in liver cancer is mainly related to recurrence and metastasis, particularly intrahepatic metastasis [16, 17]. It has been shown that filopodia formation, the previous step in cell migration, plays an important role in the metastasis of liver cancer cells [4, 5]. To determine the role of FASN in filopodia formation, we performed a filopodia localization assay in HepG2 and Huh7 liver cancer cells with different metastases. The results revealed that silencing of FASN inhibited filopodia formation in liver cancer cells, while overexpression of FASN produced the opposite effects (Fig. 2A, B). FASN has been reported to directly regulate fascin actin-bundling protein 1 (FSCN1), an important biomarker of filopodia formation [13]. Further experiments indicated that FASN positively regulated FSCN1 protein level (Fig. 2C, D). In addition, we further determined the regulation of FASN on small GTPases which also play essential roles in the regulation of filopodia formation. The results showed that silencing of FASN reduced the protein level of RAC1, CDC42, RHOA, while overexpression of FASN had the opposite effect (Fig. 2E). Since filopodia formation is necessary for cell movement, we analyzed the effects of FASN on invasion and migration of liver cancer cells. It was found that FASN also plays a positive role in the invasion and migration of liver cancer cells (Fig. 2F and Supplementary Fig. 1A–D). To confirm whether FSCN1 was involved in filopodia formation and cell movement regulated by FASN, we transfected Myc-tagged FSCN1 into FASN-silenced liver cancer cells. It was shown that overexpression of FSCN1 significantly rescued the inhibition of filopodia formation and cell invasion and migration induced by silencing of FASN (Fig. 2G, H and Supplementary Fig. 1E). These results suggest that FASN promotes filopodia formation in human liver cancer cells by regulating FSCN1.

**Fig. 3:**
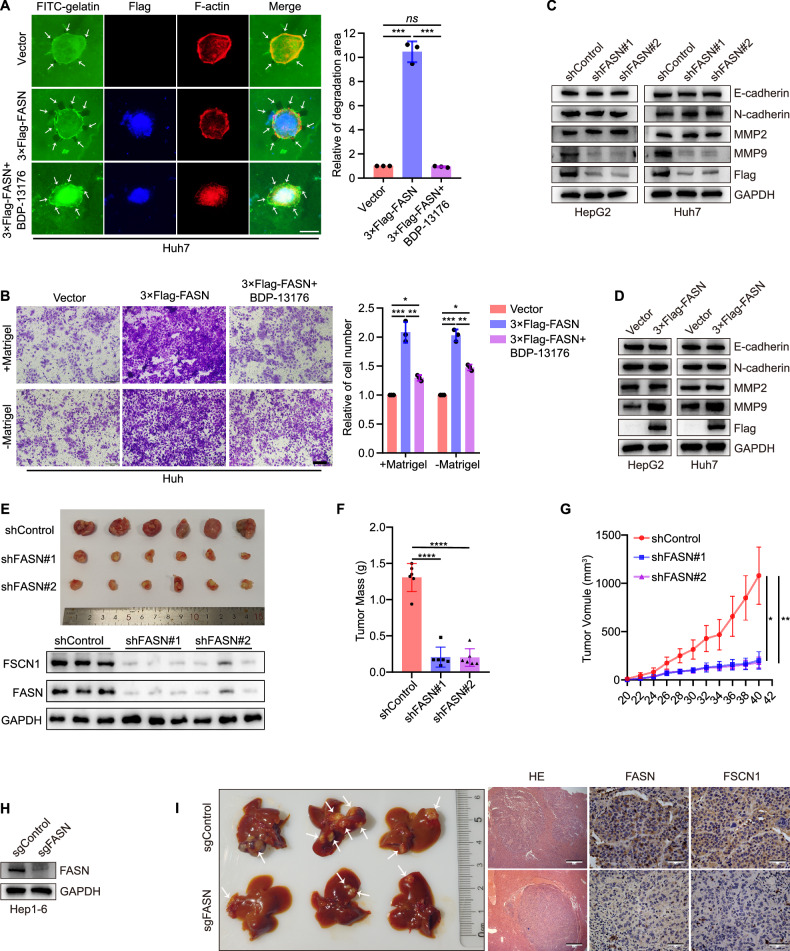
FASN promotes invasion and migration of liver cancer cells and metastasis in vivo. **A** Representative images and quantification of filopodia localization assay in Huh7 cells treated with BDP-13176 (10 μM for 24 h). Scale bar, 12.5 µm. **B** Quantification of cell invasion and migration of Huh7 cells treated with BDP-13176 (10 μM for 24 h). Scale bar, 200 µm. **C**, **D** Representative blots of E-cadherin, N-cadherin, MMP2 and MMP9 expression in HepG2 and Huh7 cells silencing or overexpressing FASN. **E** Mice tumor tissues isolated from tumors initiated with cells infected with shControl or shFASN vectors, and Representative blots of FSCN1 and FASN levels in mice tumor tissues. **F** Mice tumor mass. **G** Growth curve obtained by measuring mice tumor size on the indicated days. **H** Representative blots of FASN expression in Hep1-6 cells knock out FASN. **I** Image of livers; representative images of tissues stained with hematoxylin and eosin. Scale bar, 500 μm; representative IHC images of FASN and FSCN1 in tumor tissues. Scale bar, 50 μm. *P < 0.05, **P < 0.01, ***P < 0.001.

To further verify the role of filopodia formation on cell movement, BDP-13176, a FSCN1 inhibitor, was used in Huh7 cells overexpressed FASN. It was found that BDP-13176 could obviously restrained filopodia formation in Huh7 cells, but not completely inhibited cell invasion and migration (Fig. 3A, B). In the context of our study, we investigated the regulatory effects of FASN on epithelial-to-mesenchymal transition (EMT) and matrix metalloproteinases (MMPs). Significantly, FASN could positively regulate the protein level of MMP9, but had no effect on E-cadherin, N-cadherin, or MMP2 (Fig. 3C, D).

Furthermore, we examined the effect of FASN in a mouse xenograft model. FASN-silenced Huh7 cells or shControl Huh7 cells were transplanted subcutaneously inoculated into Balb/C nude mice. Consistent with the in vitro observations, FASN knockdown markedly inhibited tumor growth in mice, which was associated with FSCN1 (Fig. 3E–G). More precisely, Hep1-6 cells knocked out FASN were implanted beneath the liver capsule to establish an orthotopic model of liver cancer in C57BL/6J mice (Fig. 3H). It was found that knocked-out FASN obviously inhibited tumor growth and intrahepatic metastasis. The expression of FASN and FSCN1 were analyzed using immunohistochemistry (Fig. 3I). These findings suggested that FASN also acts as a tumor promoter in mice by regulating FSCN1 expression.

### The deubiquitinating enzyme complex ADRM1-UCHL5 promotes filopodia formation in liver cancer cells by stabilizing FASN

**Fig. 4:**
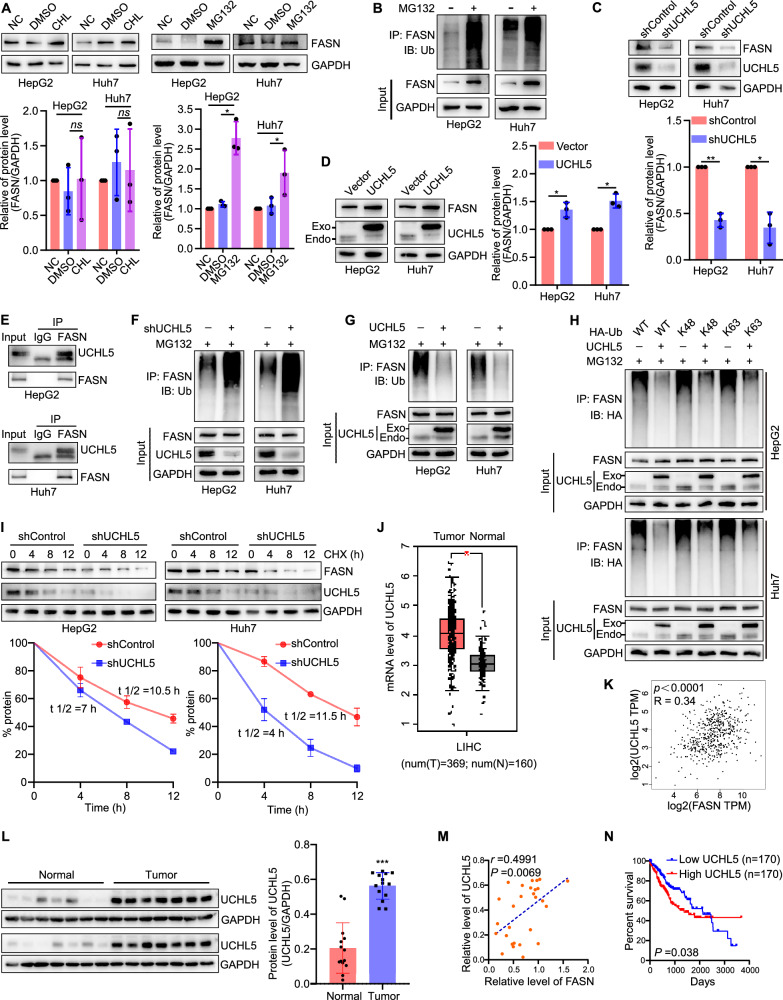
Deubiquitinating enzyme UCHL5 stabilizes FASN through reducing its K6-linked polyubiquitination. **A** Representative blots and quantification of FASN expression in liver cancer cells treated with chloroquine (CHL) or MG132. **B** Representative blots of ubiquitinated FASN in HepG2 or Huh7 cells with or without MG132. **C**, **D** Representative blots and quantification of FASN and FSCN1 expression in human liver cancer cells silencing or overexpressing UCHL5. **E** Co-immunoprecipitation assay showed that UCHL5 interacted with FASN in HepG2 and Huh7 cells. **F**, **G** Representative blots of ubiquitinated FASN in cells overexpressing or silencing UCHL5. **H** UCHL5 cleaves Lys48 and 63-linked polyubiquitin chains to deubiquitinate FASN in human liver cancer cells. **I** Representative bolts and quantification showed overexpression of UCHL5 increased the stability of FASN. **J** UCHL5 mRNA levels in human liver tumor and normal liver tissues in GEPIA (T, *n* = 369; N, *n* = 160) databases. **K** The GEPIA database revealed that the mRNA level of UCHL5 and FASN presented a direct correlation in human liver cancer. **L** Protein levels of UCHL5 in human liver tumor tissues (*n* = 14) and normal liver tissues (*n* = 14). **M** The correlation of UCHL5 expression with FASN in human normal liver tissues and liver cancer tissues. *r* = 0.4991; *P* = 0.0069. **N** Correlation between UCHL5 mRNA level and human overall survival in TCGA database (high, *n* = 170; low, *n* = 170). HepG2 and Huh7 cells were transfected with target plasmids. Twenty-four hours later, the cells were treated with MG132 (15 μM) for 8 h. *P < 0.05, **P < 0.01, ***P < 0.001.

Based on the above findings, reasons underlying the abnormal expression of FASN are worth studying. It has been reported that FASN could be degraded by ubiquitin–proteasome system (UPS), which might be a main mechanism of regulating its protein level [18–20]. Thus, we investigated the mechanisms of FASN protein degradation or stabilization in liver cancer. The results revealed that the protein level of FASN was significantly increased after the proteasomal pathway was inhibited by MG132, but not the lysosomal pathway in HepG2 and Huh7 cells (Fig. 4A). Furthermore, much more ubiquitinated FASN was observed while the proteasome function was inhibited (Fig. 4B). These results suggest that FASN can be degraded via ubiquitin–proteasome pathway in liver cancer cells.

USP14 (ubiquitin-specific peptidase 14) has been reported to directly interact with and stabilize FASN [18]. As a mammalian equivalent of USP14, the deubiquitinating enzyme UCHL5 (ubiquitin c-terminal hydrolase L5) is also associated with proteasome regulatory particles and plays important roles in proteasome modification [21–23]. Therefore, we speculated whether UCHL5 could also regulate FASN, which was consistent with the ideas from Tan et al. in the discussion [18]. To explore whether UCHL5 acts as an upstream deubiquitinating enzyme for FASN in human liver cancer cells, we first analyzed the roles of UCHL5 in the regulation of FASN protein level in HepG2 and Huh7 cells. It was found that knockdown of UCHL5 obviously inhibited the expression of FASN, while overexpression of UCHL5 had the opposite effect (Fig. 4C, D). Molecularly, the Co-IP assays showed that endogenous UCHL5 interacted with FASN in liver cancer cells (Fig. 4E). In addition, silencing of UCHL5 increased the ubiquitination level of FASN, while overexpression of UCHL5 reduced it (Fig. 4F, G). It has been reported that UCHL5 specifically cleaves K48-linked polyubiquitin chains [24, 25]. Next, to clarify whether UCHL5 could inhibit K48-linked ubiquitination of FASN, the ubiquitin mutant vectors K48 and K63, which contain arginine substitutions of all lysine residues except the one at positions 48 or 63, respectively, were used in the transfection assays. It was found that UCHL5 inhibited both K48 and K63-linked ubiquitination of FASN (Fig. 4H). Correspondingly, the protein stability of FASN was decreased upon silencing of UCHL5 (Fig. 4I). To investigate the clinical significance of UCHL5 and FASN, we first detected the mRNA level of UCHL5 in liver cancer via database. It was found that the mRNA of UCHL5 was high expressed in liver cancer (Fig. 4J). And the mRNA levels of UCHL5 and FASN presented a direct correlation in normal and liver cancer specimens (Fig. 4K). Furthermore, we collected clinical samples to determine the protein level of UCHL5 and found that the protein of UCHL5 was higher in liver cancer tissues than normal liver tissues (Fig. 4L). Correspondingly, the protein levels of UCHL5 and FASN also presented a direct correlation in normal and liver cancer specimens (Fig. 4M). Prognostically, it was found that high UCHL5 mRNA level was associated with a poor prognosis in liver cancer patients (Fig. 4N).

**Fig. 5:**
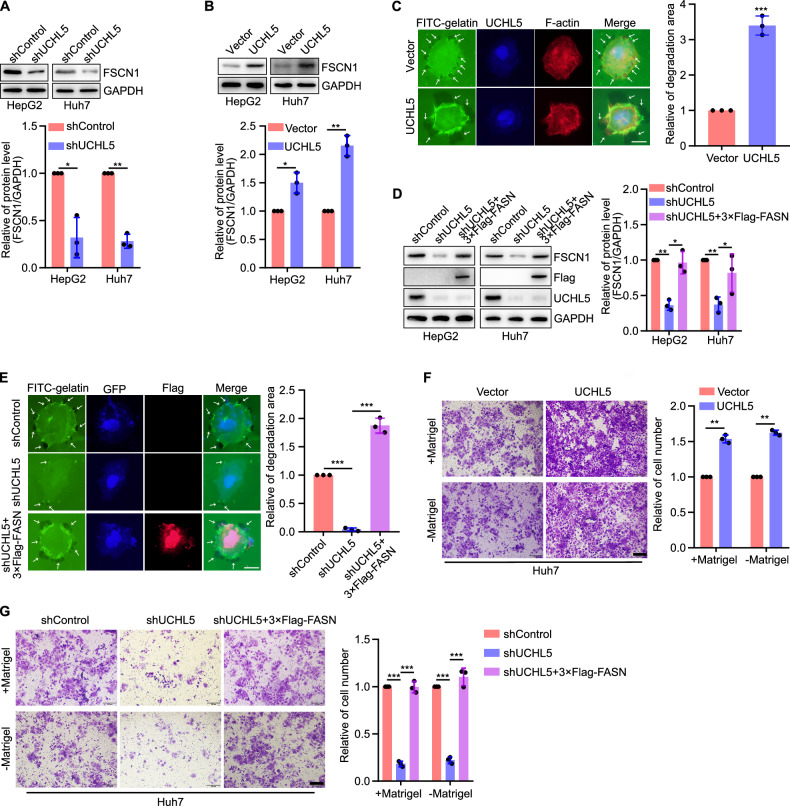
UCHL5 promotes filopodia formation in liver cancer cells by regulating FASN–FSCN1 pathway. **A**, **B** Representative blots and quantification of FSCN1 expression in human liver cancer cells silencing or overexpressing UCHL5. **C** Representative images of filopodia localization assay in Huh7 cells overexpressing UCHL5. Scale bar, 12.5 µm. **D** Representative blots and quantification of FSCN1 expression in liver cancer cells transfected with shControl, shUCHL5 and shUCHL5 + 3×Flag-FASN. **E** Representative images of filopodia localization assay in Huh7 cells transfected with shControl, shUCHL5 and shUCHL5 + 3×Flag-FASN. Scale bar, 12.5 µm. **F** Images and quantification of Huh7 cell invasion and migration while overexpressing UCHL5. Scale bar, 200 µm. **G** Images and quantification of Huh7 cell invasion and migration. Scale bar, 200 µm. *P < 0.05, **P < 0.01, ***P < 0.001.

Functionally, we determined whether UCHL5 could regulate filopodia formation in liver cancer cells. It was found that UCHL5 facilitated filopodia formation in liver cancer cells by regulating FSCN1 (Fig. 5A–C), which was associated with FASN (Fig. 5D, E). Correspondingly, UCHL5 could promote cell invasion and migration through regulating FASN (Fig. 5F, G). These results suggest that UCHL5 promotes filopodia formation in liver cancer cells by stabilizing FASN.

**Fig. 6:**
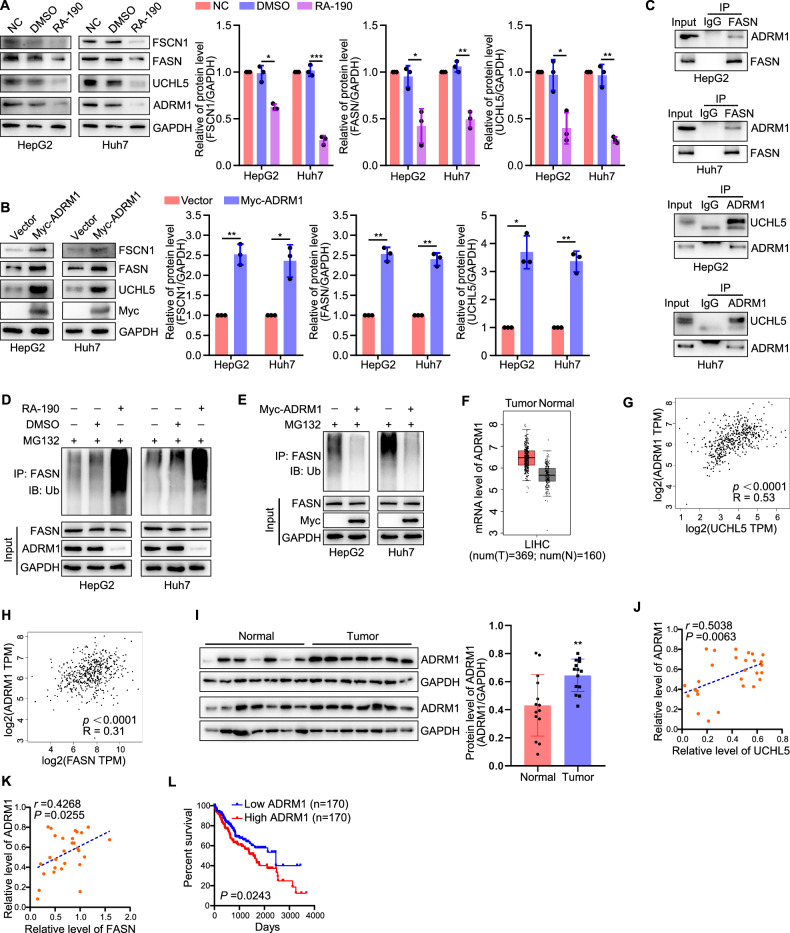
ADRM1 deubiquitinates FASN through regulating UCHL5 in liver cancer cells. **A**, **B** Representative blots and quantification of UCHL5, FASN, and FSCN1 expression in human liver cancer cells inhibiting or overexpressing ADRM1. **C** Co-immunoprecipitation assay showed that ADRM1 interacted with UCHL5 and FASN in HepG2 and Huh7 cells. **D**, **E** Representative blots of ubiquitinated FASN in human liver cancer cells inhibiting or overexpressing ADRM1. **F** ADRM1 mRNA levels in human liver tumor and normal liver tissues in GEPIA (T, *n* = 369; N, *n* = 160) databases. **G** The GEPIA database revealed that the mRNA level of ADRM1 and UCHL5 presented a direct correlation in human liver cancer. **H** The GEPIA database revealed that the mRNA level of ADRM1 and FASN presented a direct correlation in human liver cancer. **I** Protein levels of ADRM1 in liver tumor tissues (*n* = 14) and normal liver tissues (*n* = 14). **J** The correlation of ADRM1 expression with UCHL5 in human normal liver tissues and liver cancer tissues. *r* = 0.5038; *P* = 0.0063. **K** The correlation of ADRM1 expression with FASN in human normal liver tissues and liver cancer tissues. *r* = 0.4268; *P* = 0.0255. **L** Correlation between ADRM1 mRNA level and human overall survival in TCGA database (high, *n* = 170; low, *n* = 170). HepG2 and Huh7 cells were transfected with target plasmids. Twenty-four hours later, the cells were treated with MG132 (15 μM) for 8 h. *P < 0.05, **P < 0.01, ***P < 0.001.

Since UCHL5 exerts its role as a deubiquitinating enzyme through recruitment and activation by ADRM1 (adhesion regulating molecule 1), also known as Rpn13 [26, 27], we further explored the regulation and mechanism of action of ADRM1 on FASN in liver cancer. Briefly, overexpression of ADRM1 promoted the expression of UCHL5, as well as FASN and FSCN1, while inhibition of ADRM1 had the opposite effect (Fig. 6A, B). Next, the Co-IP assays revealed that endogenous ADRM1 interacted with UCHL5 and FASN in HepG2 and Huh7 cells (Fig. 6C). Furthermore, inhibition of ADRM1 increased the ubiquitination level of FASN, while overexpression of ADRM1 reduced it (Fig. 6D, E). Clinically, the mRNA of ADRM1 was high expressed in liver cancer (Fig. 6F). And the mRNA levels of ADRM1 and UCHL5 presented a direct correlation in normal and liver cancer specimens, as well as ADRM1 and FASN (Fig. 6G, H). Proteinally, the protein of ADRM1 was higher in liver cancer tissues than normal liver tissues (Fig. 6I). And both the protein levels of ADRM1-UCHL5 and ADRM1-FASN presented a direct correlation in normal and liver cancer specimens (Fig. 6J, K). Prognostically, high ADRM1 mRNA level was associated with a poor prognosis in liver cancer patients (Fig. 6L). These results suggest that ADRM1-UCHL5 could promote the deubiquitination of FASN and there is a direct correlation between them in clinical expression.

### The E3 ubiquitin ligase SIAH1 destabilizes ADRM1 by promoting its K6‑linked polyubiquitination and proteasome degradation in liver cancer cells

**Fig. 7:**
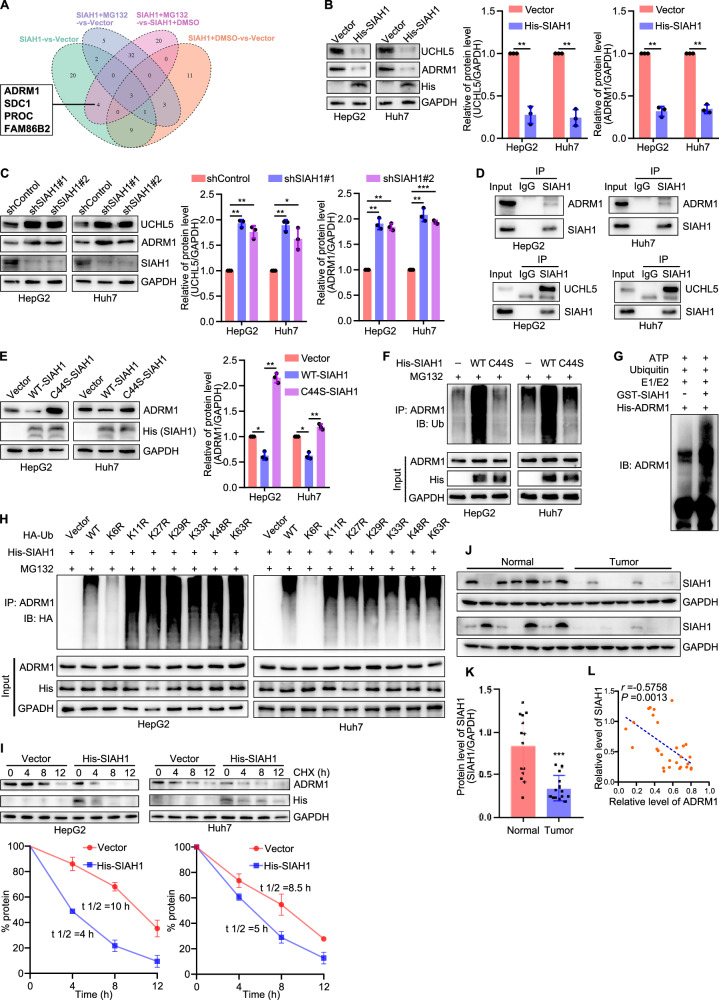
The E3 ligase SIAH1 interacts with and ubiquitinates ADRM1 to promote its degradation in liver cancer cells. **A** TMT proteomics analysis comparing protein expression of empty-vector and overexpression of SIAH1 (−/ + MG132) in HepG2 cells. **B**, **C** Representative blots and quantification of ADRM1 and UCHL5 expression in human liver cancer cells overexpressing or silencing SIAH1. **D** Co-immunoprecipitation assay showed that SIAH1 interacted with ADRM1 in HepG2 and Huh7 cells. **E** Representative blots and quantification of ADRM1 expression in human liver cancer cells with a SIAH1 mutation. **F** Representative blots of ubiquitinated ADRM1 in human liver cancer cells with a SIAH1 mutation. **G** Representative blots of ubiquitination of FASN by SIAH1 in vitro. **H** SIAH1 ubiquitinated FASN through Lys33-linked ubiquitin chains. **I** Representative bolts and quantification showed overexpression of SIAH1 decreased the stability of ADRM1. **J** Representative blots and, **K** quantification of SIAH1 in human liver tumor tissues (*n* = 14) and normal liver tissues (*n* = 14). **L** The correlation of SIAH1 expression with ADRM1 in human normal liver tissues and liver cancer tissues. *r* = −0.5758; *P* = 0.0013. HepG2 and Huh7 cells were transfected with target plasmids. Twenty-four hours later, the cells were treated with MG132 (15 μM) for 8 h. *P < 0.05, **P < 0.01, ***P < 0.001.

SIAH1 (Seven in absentia homolog 1), a ubiquitin ligase, has been reported to be involved in cell cycle, apoptosis, DNA damage repair, and hypoxia stress [28–30]. Oncologically, studies showed that SIAH1 was associated with the involvement of liver cancer [31–33]. In a preliminary study, we performed a TMT proteomics analysis in HepG2 cells to compare the protein expression between empty-vector and overexpression of SIAH1 (−/ + MG132) and found that ADRM1 was one of the differential proteins (Fig. 7A). Subsequent experiments found that SIAH1 could degrade ADRM1 through the UPS pathway but not the lysosomal pathway in HepG2 and Huh7 cells (Supplementary Fig. 2).

Furthermore, we analyzed the regulatory effect of SIAH1 on the ADRM1-UCHL5 pathway. The results showed that overexpression of SIAH1 decreased the expression of ADRM1 and UCHL5, while silencing of SIAH1 had the opposite effect (Fig. 7B, C). To explore the mechanism by which SIAH1 degrades ADRM1, we first determined the interaction between them in liver cancer cells. The Co-IP assays showed that SIAH1 interacted with ADRM1 in HepG2 and Huh7 cells, as well as UCHL5 (Fig. 7D). It has been reported that the RING domain of SIAH1 is required for its ubiquitin ligase activity [34]. To investigate whether SIAH1 E3 ligase activity is required for SIAH1-induced ADRM1 protein degradation, we generated a SIAH1-RING mutant in which Cys44 in the RING domain was converted to Serine (C44S-SIAH1). Previous studies revealed that this mutant has no E3 ligase activity [35]. Our results showed that, compared with wild-type SIAH1 (WT-SIAH1), the SIAH1-RING mutant largely abolished the ability of SIAH1 to degrade ADRM1 (Fig. 7E). In addition, compared with the wildtype SIAH1, SIAH1-RING mutant also largely abolished SIAH1-mediated ADRM1 ubiquitination (Fig. 7F). Furthermore, we confirmed the direct ubiquitination of SIAH1 on ADRM1 using recombinant proteins to simulate an in vitro environment in a test tube (Fig. 7G). In addition, we observed that SIAH1-mediated polyubiquitination of ADRM1 was initiated by K6-linked chains in HepG2 and Huh7 cells, because ubiquitination of ADRM1 is almost undetectable when expressing the K6R ubiquitin mutant (Fig. 7H). We also demonstrated that the protein stability of ADRM1 was decreased upon upregulation of SIAH1 (Fig. 7I).

Clinically, SIAH1 was found to be downregulated in liver cancer samples (Fig. 7J, K), and there was a directly negative correlation between SIAH1 and ADRM1 protein levels in normal and liver cancer specimens (Fig. 7L). These results suggest that K6-linked chains serve as the principal ubiquitin chain type for SIAH1-induced ADRM1 proteasomal degradation and there is a direct negative correlation between them in clinical expression.

### SIAH1 directly promotes K33‑linked polyubiquitination and degradation of FASN, thereby inhibiting filopodia formation in liver cancer cells

**Fig. 8:**
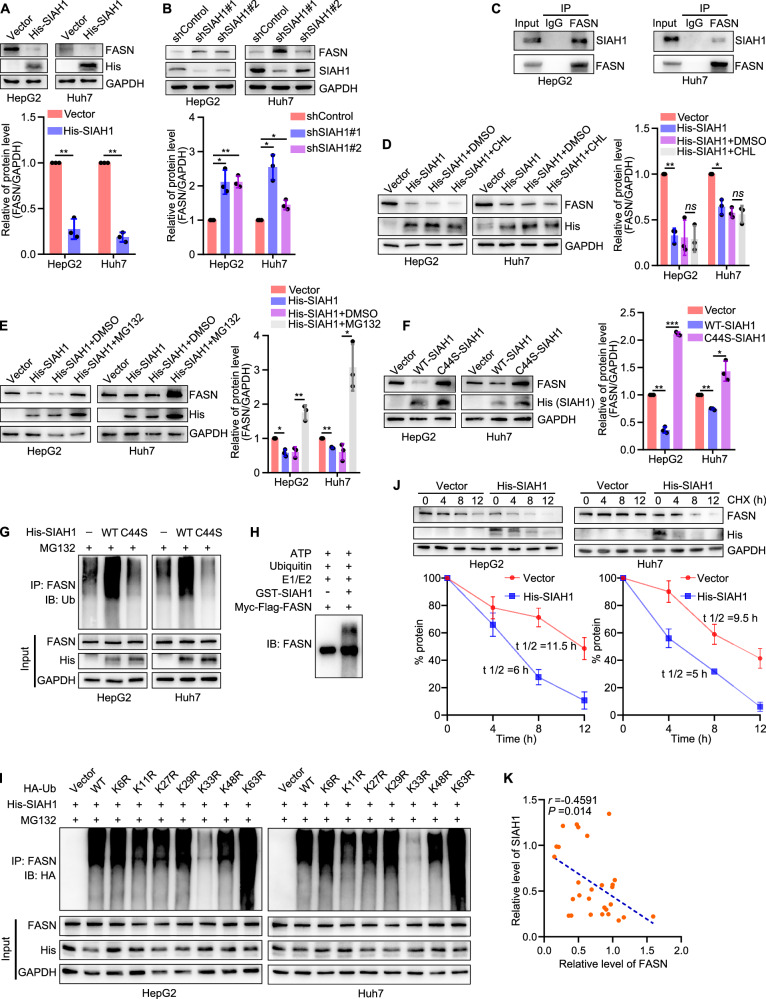
SIAH1 directly ubiquitinates FASN to promote its degradation in liver cancer cells. **A**, **B** Representative blots and quantification of FASN expression in human liver cancer cells overexpressing or silencing SIAH1. **C** Co-immunoprecipitation assay showed that SIAH1 interacted with FASN in HepG2 and Huh7 cells. **D** Representative bolts and quantification of FASN expression in liver cancer cells transfected with His-SIAH1 plasmid (+CHL). **E** Representative blots and quantification showed that MG132 could block the degradation of FASN in SIAH1-upregulated cells. **F** Representative blots and quantification of FASN expression in human liver cancer cells with a SIAH1 mutation. **G** Representative blots of ubiquitinated FASN in human liver cancer cells with a SIAH1 mutation. **H** Representative blots of ubiquitination of FASN by SIAH1 in vitro. **I** SIAH1 ubiquitinated FASN through Lys33-linked ubiquitin chains in HepG2 and Huh7 cells. **J** Representative bolts and quantification showed overexpression of SIAH1 decreased the stability of FASN. **K** The correlation of SIAH1 expression with ADRM1 in human normal liver tissues and liver cancer tissues. *r* = −0.4591; *P* = 0.014. HepG2 and Huh7 cells were transfected with target plasmids. Twenty-four hours later, the cells were treated with MG132 (15 μM) for 8 h. *P < 0.05, **P < 0.01, ***P < 0.001.

The above results have shown that SIAH1 acts as a ubiquitin ligase toward ADRM1. This can affect the recruitment of UCHL5, thereby regulating the protein stability of FASN. Therefore, we asked whether SIAH1 directly regulates FASN through the ubiquitination pathway. To address this question, we analyzed the protein level of FASN in liver cancer cells when SIAH1 was overexpressed or silenced and found that overexpression of SIAH1 decreased the expression of FASN, while knockdown of SIAH1 increased it (Fig. 8A, B). Mechanistically, SIAH1 was demonstrated to interact with FASN in HepG2 and Huh7 cells (Fig. 8C). In addition, MG132, but not CHL, could block the degradation of FASN in SIAH1-upregulated cells (Fig. 8D, E). The protein level of FASN could be restored while the ubiquitin ligase ability of SIAH1 was defective (Fig. 8F). Moreover, compared with wildtype SIAH1, the ubiquitination level of FASN in HepG2 and Huh7 cells was significantly decreased when SIAH1 ubiquitin ligase activity was inactivated (Fig. 8G). More importantly, we found that SIAH1 could directly promote FASN ubiquitination in vitro environment (Fig. 8H). We further observed that SIAH1-mediated FASN polyubiquitination was initiated by K33-linked chains in HepG2 and Huh7 cells (Fig. 8I). Similarly, we determined that the protein stability of FASN was decreased in SIAH1-upregulated cells (Fig. 8J). In terms of clinical protein expression correlation, the protein levels of SIAH1 and FASN exhibited a directly negative correlation in normal and liver cancer specimens (Fig. 8K).

**Fig. 9:**
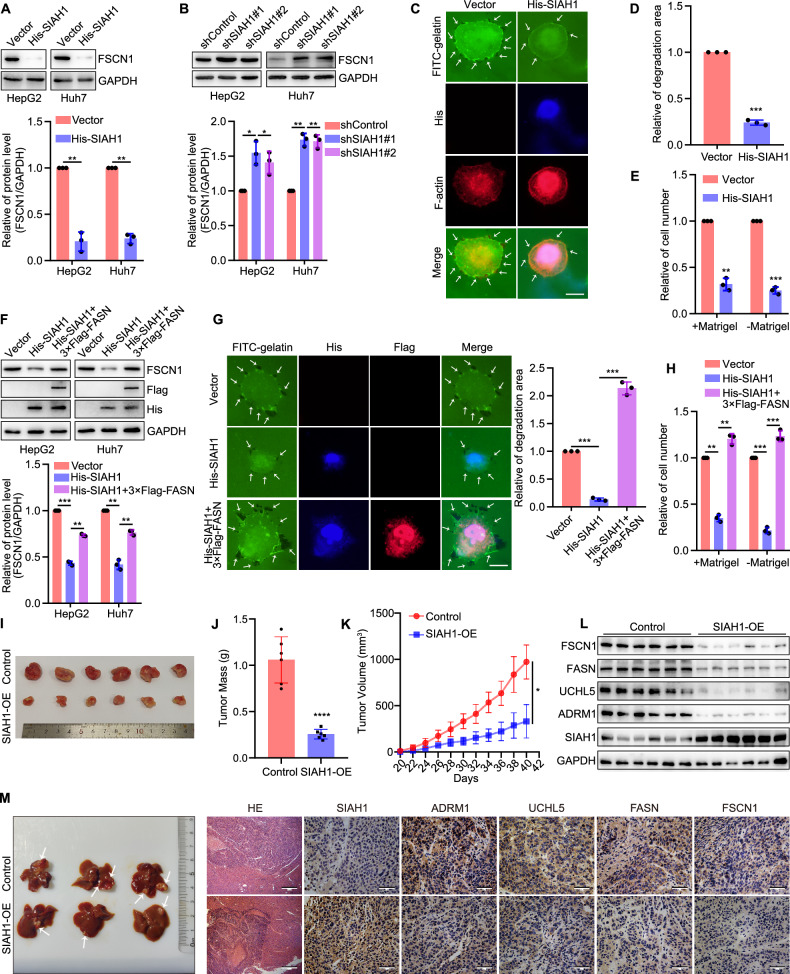
SIAH1 inhibits filopodia formation in liver cancer cells by regulating the FASN–FSCN1 pathway. **A**, **B** Representative blots and quantification of FSCN1 expression in liver cancer cells overexpressing or silencing SIAH1. **C**, **D** Representative images of filopodia localization assay in Huh7 cells overexpressing SIAH1. Scale bar, 12.5 µm. **E** Quantification of Huh7 cell invasion and migration while overexpressing SIAH1. **F** Representative blots and quantification showed that overexpression of FASN could block the degradation of FSCN1 in SIAH1-upregulated cells. **G** Representative images of filopodia localization assay of Vector, His-SIAH1, and His-SIAH1 + 3×Flag-FASN groups in Huh7 cells. Scale bar, 12.5 µm. **H** Quantification of Huh7 cell invasion and migration. **I** Mice tumor tissues isolated from tumors initiated with cells infected with Control or SIAH1 OE (SIAH1 overexpression) vectors. **J** Tumor mass. **K** Growth curve obtained by measuring tumor size on the indicated days. **L** Representative blots of protein levels in tumor tissues. **M** Image of livers; representative images of tissues stained with hematoxylin and eosin. Scale bar, 500 μm; representative IHC images of SIAH1, ADRM1, UCHL5, FASN, and FSCN1 in tumor tissues. Scale bar, 50 μm. *P < 0.05, **P < 0.01, ***P < 0.001, ****P < 0.0001.

To confirm whether SIAH1 affects filopodia formation in liver cancer cells by regulating FASN, we detected the regulation of FSCN1 by SIAH1. It was found that overexpression of SIAH1 significantly decreased the expression of FSCN1 in HepG2 and Huh7 cells, while silencing of SIAH1 increased it (Fig. 9A, B). Morphologically, overexpression of SIAH1 inhibited filopodia formation in Huh7 cells, as well as cell invasion and migration (Fig. 9C–E and Supplementary Fig. 3A). We further performed rescue experiments to confirm the above findings by overexpressing 3×Flag-FASN in SIAH1-upregulated liver cancer cells. It was shown that overexpression of FASN effectively rescued the FSCN1 expression induced by overexpression of SIAH1 (Fig. 9F), and restored Huh7 cell filopodia formation and movement (Fig. 9G, H and Supplementary Fig. 3B). These results suggest that SIAH1 regulates filopodia formation in human liver cancer cells by mediating K33-linked polyubiquitination and proteasomal degradation of FASN.

To explore the association between SIAH1 and the growth and metastasis of liver cancer in vivo, Huh7 cells overexpression of SIAH1 were subcutaneously injected into Balb/c nude mice (Fig. 9I). Predictably, the mice tumor growth was significantly inhibited upon SIAH1 was overexpressed (Fig. 9J, K). The expression of proteins was detected by western blotting (Fig. 9L). Furthermore, an orthotopic model of liver cancer was established using Hep1-6 overexpressed SIAH1. Predictably, images of the liver and H.E. staining showed that overexpression of SIAH1 inhibited tumor growth and intrahepatic metastasis, and immunohistochemistry was used to analyze the expression of SIAH1, ADRM1, UCHL5, FASN, and FSCN1 (Fig. 9M). Collectively, these results revealed that SIAH1 acts as a tumor suppressor in mice, and loss of SIAH1 may be an important event during the development and progression of liver cancer in mice.

## Discussion

Multiple studies have confirmed the important roles of ubiquitination and deubiquitination in the occurrence and development of tumors, such as proteasomal degradation, selective autophagy, cell signaling regulation, endocytosis, and receptor trafficking, DNA damage response, cell cycle control, and programmed cell death [36–39]. In this study, we found SIAH1 is lower expressed in liver cancer. On the one hand, low expression of SIAH1 can trigger FASN to undergo deubiquitination and escape from proteasomal degradation through ADRM1-UCHL5 complex. On the other hand, loss of SIAH1 can directly weaken the ubiquitination of FASN, leading to FASN protein accumulation. These findings not only reveal a novel mechanism of SIAH1-mediated liver cancer occurrence and progression but also provide a certain theoretical basis for the treatment of liver cancer.

Initially, tumor cell migration begins with the formation of protuberances, in which filopodia play a leading role [40]. Although studies have shown that the formation of filopodia plays an important role in the migration of liver cancer cells [4, 5], the effect and mechanism of filopodia in liver cancer still require further exploration. Recent studies revealed that FASN directly regulates FSCN1 which participates in the formation of filamentous pseudopodia, lamellar pseudopodia, and microspines and the coding of cytoskeletal proteins, thus promoting the migration and invasion of liver cancer cells [12, 13]. FASN is a key enzyme required for the synthesis of fatty acids and some biologically important lipid precursors, thereby regulating metabolism, cell survival and proliferation, DNA replication and transcription, and protein degradation by catalyzing the generation of endogenous fatty acids and interacting with various cancer control networks [6–8]. It has been shown that FASN can promote the metastasis of liver cancer cells, and its overexpression is closely related to clinical invasiveness and poor prognosis [10, 11], which was also observed in this study both in human and mice. Although studies on FASN mostly focus on its regulation of lipid metabolism, its promotion of liver cancer transfer is not entirely dependent on it [9, 13], and targeting FASN as a monotherapy has still shown limited efficacy [41]. Therefore, directly exploring the mechanism of FASN regulating cell migration, such as filopodia formation, may provide new insights. In our study, we confirmed that FASN could positively regulate FSCN1 and determined that FASN could promote filopodia formation in human liver cancer cells by regulating FSCN1. In addition, we further found that some small GTPases, such as CDC42, RAC1 and RHOA, were also regulated by FASN. These results suggest that FASN promotes the filopodia formation in liver cancer cells through regulating multiple downstream targets. Interestingly, when investigating the correlation between FASN regulation of filopodia formation and invasion and migration of liver cancer cells, we observed that inhibiting filopodia formation did not completely impede cell motility. Additionally, we demonstrated that FASN can impact MMP9 expression, but does not exert a significant influence on epithelial-mesenchymal transition (EMT). The evidence suggests that FASN can impact the metastasis of liver cancer through various pathways. Based on these findings, it is reasonable to further explore the regulatory mechanism of high expression of FASN in liver cancer.

Multiple reports showed that FASN could be regulated by UPS [18–20], which was also observed in our results. Therefore, this study focused on identifying UPS-related enzymes that regulate FASN. Studies have shown that USP14 is a specific upstream DUB of FASN and speculated that UCHL5 may have the same effect [18]. UCHL5 is a member of the UCH family and can function as DUB in combination with proteasome. The c-terminal deubiquitinating enzyme adapter of ADRM1 binds to and activates UCHL5, and then reverses the ubiquitination of some key substrates and maintains protein stability [26, 27]. In this study, we determined that ADRM1-activated UCHL5 was upregulated in liver cancer and stabilized FASN through deubiquitinating it, thereby promoting the expression of FSCN1 and filopodia formation in human liver cancer cells, as well as cell movement. Correspondingly, as the UCHL5-specific upstream activator, ADRM1 also plays a similar role.

SIAH1 is a highly conserved E3 ubiquitin ligase that can regulate transcription factors, neurotransmitters, hypoxia-inducible factors, and other substrates and then affect several cell life activities, such as cell cycle, apoptosis, DNA damage repair, and hypoxia stress [28–30]. Studies have shown that SIAH1 plays an important role in the occurrence and development of liver cancer [31–33]. In this study, we found that SIAH1 could inhibit the expression of UCHL5 through ubiquitinating and degrading ADRM1. It is speculated that SIAH1 may also be involved in the regulation of the FASN–FSCN1 pathway and filopodia formation in human liver cancer cells. To confirm this concept, we examined the regulation of FASN–FSCN1 pathway and filopodia formation in liver cancer cells by manipulating SIAH1. The results showed that SIAH1 could decrease the expression of FSCN1 by directly ubiquitinating FASN, thereby inhibiting filopodia formation in human liver cancer cells, as well as cell invasion and migration.

Polyubiquitination is mediated by seven lysines, including K6, K11, K27, K29, K33, K48, and K63. While K48- and K63-linked chains are broadly covered in the literature, the other types of chains assembled through K6, K11, K27, K29, and K33 residues deserve equal attention considering the latest discoveries [42]. In this study, we redefined the role of partial lysine residues in substrate degradation in human liver cancer cells. We found that ADRM1 polyubiquitination mediated by SIAH1 was initiated by K6-linked chains, while FASN was initiated by K33-linked chains. This could provide a basis for the study of non‑canonical protein ubiquitination.

**Fig. 10:**
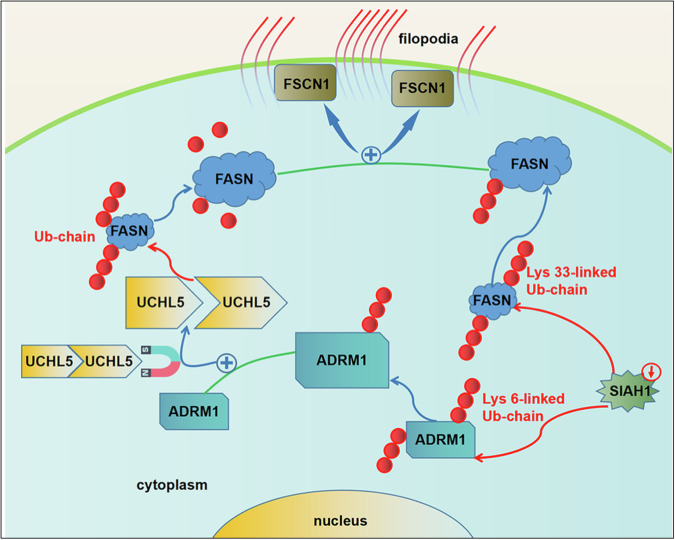
Schematic illustration of this study.

In conclusion, the current study identified that FASN is upregulated in liver cancer and then promotes filopodia formation and metastasis of liver cancer cells by regulating FSCN1 and other pathways. Molecularly, we identified that the upregulation of FASN is caused by the increment of the deubiquitination enzyme UCHL5. In this regard, low expression of SIAH1 decreases the ubiquitination and degradation of ADRM1 thus increasing its protein level, which further recruits and activates the deubiquitination enzyme UCHL5, ultimately makes FASN to undergo deubiquitination and escapes from proteasomal degradation. Additionally, we observed that the accumulation of FASN is also related to its low level of ubiquitination, where SIAH1 acts as a ubiquitin ligase towards FASN, and low expression of SIAH1 reduces the ubiquitination and degradation of FASN. Both the two pathways participate in the regulation of FASN in liver cancer (Fig. 10). Importantly, the correlation of SIAH1-FASN–FSCN1 axis also verified by human clinical tissues and mice model. Our study has demonstrated the oncogenic effect and regulatory mechanism of FASN in liver cancer, which will provide new insights for further study of the molecular mechanism of liver cancer metastasis and molecular targeted therapy for liver cancer.

## Methods

### Tissues

Patients who were pathologically diagnosed with liver cancer after surgery at the Affiliated Hospital of Xuzhou Medical University were included. Normal liver specimens were obtained from patients undergoing partial hepatectomy to treat liver rupture due to trauma. All tissue samples were immediately frozen in liquid nitrogen and stored at −80 °C.

### Animal survival model

All the animal experimental protocols were approved by the Animal Care and Use Committee at the Xuzhou Medical University. Animal maintenance was in accordance with the Animal Experiment Center of Xuzhou Medical University standard guidelines. The protocols were performed in accordance with the Guide for the Care and Use of Laboratory Animals published by the National Institutes of Health. The Huh7 cells (1 × 10^6^ cells) were resuspended in 100 μL PBS and then were injected subcutaneously into 4-week-old Balb/c nude mice to establish the mouse xenograft model (6 male mice/group). The Hep1-6 cells (10^6^/20 μL) were then injected beneath the liver capsule of 8-week-old C57BL/6J mice to establish the orthotopic model of liver cancer (3 male mice/group). Each group of animals was randomly assigned.

### Western blotting (WB) analysis

WB was performed as previously described in our published articles [43, 44]. For western blotting, cells were lysed in RIPA buffer supplemented with a protease inhibitor cocktail and centrifuged at 12,000×*g* at 4 °C for 10 min; equal amounts of protein were subjected to 8% SDS-PAGE and then transferred onto a 0.45-micrometer pore size PVDF membrane. After blocking with 3% bovine serum albumin, the membrane was incubated overnight with the primary antibodies (FASN, FSCN1, UCHL5, ADRM1, SIAH1, HA, Flag, Myc, His, Ub, GAPDH, etc.) at 4 °C and then with secondary antibodies for 1 h at room temperature. After washing with 1×TBST, ECL Plus western blotting Substrate was used to detect the protein bands, and a chemiluminescence detection system was used for visualization. ImageJ 1.8.0 was used to quantify band density. Relative protein levels were determined by normalizing the optical density values of the target protein with those of the loading control. The antibodies used are as Supplementary Table 1.

### Cell culture

The 293T cell line and the liver cancer cell lines HepG2 and Huh7 were provided by the Stem Cell Bank, Chinese Academy of Sciences (Shanghai, China), and were cultured in an incubator at 37 °C and 5% CO_2_ with Minimum Essential Medium (MEM) or Dulbecco’s Modified Eagle Medium (DMEM) (Yuanpei, Shanghai, China) supplemented with 10% fetal bovine serum (FBS, Gibco, Shanghai, China). All cells were certified by SRT.

### Lentivirus construction and transfection

To produce the lentiviruses, 293T cells were cotransfected with the corresponding plasmids (shControl, shFASN, shUCHL5 and shSIAH1) and helper plasmids (psPAX2 and pMD2.G) using Hieff Trans™ Liposomal Transfection Reagent (Yeasen, Shanghai, China). After 72 h, the lentiviruses were collected and subsequently used to infect HepG2 and Huh7 cells. Forty-eight hours after infection, the cells were continuously cultured in a medium containing 2.5 μg/mL puromycin (Beyotime). The surviving cells were cultured into cell lines stably expressing shControl, shFASN, shUCHL5, and shSIAH1. The primer pairs used are shown in Supplementary Table 2.

### Filopodia localization assay

The cell climbing pieces in a 24-well plate were covered with FITC-gelatin (Biovision, San Francisco, USA) for 30 min and fixed with 100 µL precooled glutaraldehyde (0.5%) for 30 min. After washing with PBS, the cell climbing pieces were incubated with 1 mL precooled sodium borohydride (5 mg/mL) and then washed with PBS again. After alcohol (70%) disinfection for 30 min, aldehyde quenching with serum-free medium at 37 °C was undertaken for 1 h. Subsequently, the cells were placed on the 24-well plate (5 × 10^4^ cells/well) and cultured in an incubator at 37 °C. Five hours later, the cells were fixed with 4% paraformaldehyde, permeabilized with 0.5% Triton X-100, and blocked with 5% non-fat milk. The cells were incubated with primary antibodies at 4 °C overnight and fluorescent secondary antibodies at 25 °C for 1 h in the dark. They were then incubated with phalloidine (Yeasen) at 25 °C for 30 min in the dark. Finally, the anti-quenching agent was used to seal the tablet, and imaging was performed using a fluorescence microscope (IX71; Olympus, Tokyo, Japan).

### Co-immunoprecipitation (Co-IP) assay

The HepG2 and Huh7 cells were lysed with ice-cold IP buffer (1% Triton X-100, 150 mM NaCl, 20 mM HEPES, 2 mM EDTA, 5 mM MgCl_2_, pH 7.4). The cell lysates containing the proteins were conjugated to the beads after being incubated overnight with the indicated antibodies. Subsequently, the beads were eluted and subjected to WB assays using the indicated primary and corresponding secondary antibodies.

### Ubiquitination assay in vivo

The HepG2 and Huh7 cells were successfully transfected with the desired plasmids and then lysed with ice-cold IP buffer. Similarly, the cell lysates containing the proteins were conjugated to the beads after being incubated overnight with the indicated antibodies. The beads were eluted and subjected to WB assays using the anti-HA/Ub antibodies and corresponding secondary antibodies.

### Ubiquitination assay in vitro

Ubiquitination assay in vitro was performed using a kit (Bio-Techne, Minnesota, USA) according to the manufacturer's instructions. A 20 μL reaction mixture was prepared in a 1.5-mL polypropylene tube using the following volumes: 2 µL 10×Reaction Buffer, 2 µL 10×Ubiquitin, 1 µL 20×E1 Enzyme, 2 μL 10×E2 conjugating enzyme (Bio-Techne), 4 μL (contain 2 µg) E3 Ligase enzyme (LSBio, Shanghai, China), 4 μL (contain 2 µg) substrate protein (LSBio), 2 µL 10×Mg^2+^-ATP solution, 3 μL ddH_2_O was used to supplement 20 μL of total reaction volume. The group without E3 was used as a negative control. After slightly mixing, the tubes were incubated for 1.5 h in 37 °C water bath. Reactions were terminated with DTT (10 mM, Bio-Techne) and then analyzed via SDS-PAGE gel. WB with an anti-substrate antibody was used to determine conjugate formation, which may appear as either a high-molecular-weight smear or a discrete banding pattern.

### Quantitative TMT-based proteomic analysis

Quantitative TMT-based proteomic analysis was performed by Frasergen Bioinformatics Co. Ltd (Wuhan, Hubei, China). Total protein was extracted from HepG2 (Vector, SIAH1, SIAH1 + DMSO and SIAH1 + MG132) cells. Each protein (100 μg) was denatured in 8 mol/L urea in 50 mmol/L NH_4_HCO_3_ (pH 7.4) and alkylated with 10 mmol/L iodoacetamide for 1 h at 37 °C. Then each sample was diluted tenfold with 25 mmol/L NH_4_HCO_3_ and digested with trypsin at a ratio of 1:100 (trypsin/substrate) for 6 h at 37 °C. A 25 μg aliquot of digested peptides for each sample was subjected to eight-plex TMT labeling according to the manufacturer’s instructions. Peptides from each TMT experiment was subjected to capillary liquid chromatography-tandem mass spectrometry (LC-MS/MS) using a Q Exactive Hybrid Quadrupole-Orbitrap Mass Spectrometer (Thermo Fisher Scientific, Waltham, MA, USA). The quantitative analysis was conducted by calculating the ratios between the experimental and control groups. The TMT experiment was repeated three times. The changes were considered significant if the change value increased or decreased by >1.5 fold, and the *P* value was <0.05. The original mass spectrum data were searched by database using Mascot 2.2 and Proteome Discoverer 1.4 (Thermo Fisher Scientific, Waltham, MA, USA).

### Establishment of the mouse xenograft model

The animal studies have been conducted in accordance with the Institutional Animal Care and Use Committee of Xuzhou Medical University. Six male nude mice (Vital River Laboratory Animal Technology, Beijing, China) per group with no significant difference in body weight (at 4 weeks of age) were reared in a sterile environment for 1 week. After disinfecting their skin, 100 µL of Huh7 cell suspension (10^6^/mL) was injected into the dorsal side of the right hind limb. The mice were then returned to their cages and housed under the same conditions. Tumor length and width were measured every other day using Vernier calipers, and tumor volume was calculated as follows: length × (width^2^/2). After 6 weeks, all mice were euthanized, and their subcutaneous tumors were removed and then all tumors were isolated for protein extraction.

### Survival model of nude mice

Twenty-five male nude mice per group with no significant difference in body weight (at 4 weeks of age) were reared in a sterile environment for 1 week. After disinfecting their tail, 100 µL of Huh7 cell suspension (10^6^/mL) was injected into the caudal vein. The mice were then returned to their cages and housed under the same conditions until they die of natural causes. Time of death was recorded and survival curve was drawn.

### Establishment of the orthotopic model of liver cancer

The animal studies have been conducted in accordance with the Institutional Animal Care and Use Committee of Xuzhou Medical University. Three male C57BL/6J mice (Vital River Laboratory Animal Technology, Beijing, China) per group with no significant difference in body weight (at 7 weeks of age) were reared in a sterile environment for 1 week. After a 12-h fast, the abdomen was opened under conventional anesthesia to expose the left lobe of the liver. The Hep1-6 cells (10^6^/20 μL) were then injected beneath the liver capsule. Gentle pressure was applied to the puncture site with a cotton swab to prevent leakage. After the closure of the abdominal cavity, it was maintained at 37 °C for 2 h before regular feeding commenced. Two weeks later, an open liver was photographed and subsequently subjected to follow-up analysis using H.E. staining and immunohistochemistry.

### Transwell migration and invasion assays

Transwell migration and invasion assays were carried out as described and adjusted [43, 45, 46]. Briefly, transwell chambers (24-well, 8.0-µm pore membranes, NY) were used in the migration assay. In total, 1 × 10^4^ cells/well were seeded in the upper chamber in 200 µL of serum-free medium, and 600 µL complete medium as a chemoattractant was added in the lower chamer. After incubated for 48 h (HepG2) or 24 h (Huh7) at 37 °C, some cells successfully passed through the upper chamber membrane, and the cells on the lower surface of the membrane are the migrated sells. After fixed with 4% paraformaldehyde for 30 min and stained with 0.3% crystal violet for 30 min, the migrated cells were photographed by an inverted microscope.

The transwell invasion assay was conducted as described above, except that 100 µl of 1×Matrigel (BD, China, Shanghai; phosphate-buffered saline [PBS] was used for dilution on ice) was added to the upper compartment. Three random fields of view in each chamber were selected for counting. The control group was labeled as ‘1’ for statistical purposes.

### Statistical analysis

Data represent the results of experiments repeated at least three times, and all quantitative data are expressed as the mean ± SD. Statistical analysis was performed using GraphPad Prism (v.10.0; GraphPad Software, USA). Student’s *t* tests were used to compare samples with normality, homogeneity of variance, and independence. Nonparametric tests were used to analyze measurement or count data that did not meet these requirements. *P* < 0.05 was considered statistically significant.

### Supplementary information

Supplementary Figures

Supplementary materials

## Data Availability

All data supporting the findings of this study are available from the corresponding author on reasonable request.
