# Supplementary material for: Deficiency of SIAH1 promotes the formation of filopodia by increasing the accumulation of FASN in liver cancer
Source: Cell Death Dis. 2024 Jul 29;15(7):537. doi: 10.1038/s41419-024-06929-7 (PMC11286965; doi:10.1038/s41419-024-06929-7)
Supplement: Supplementary file 1 — Supplementary Figures [file 41419_2024_6929_MOESM1_ESM.pdf]

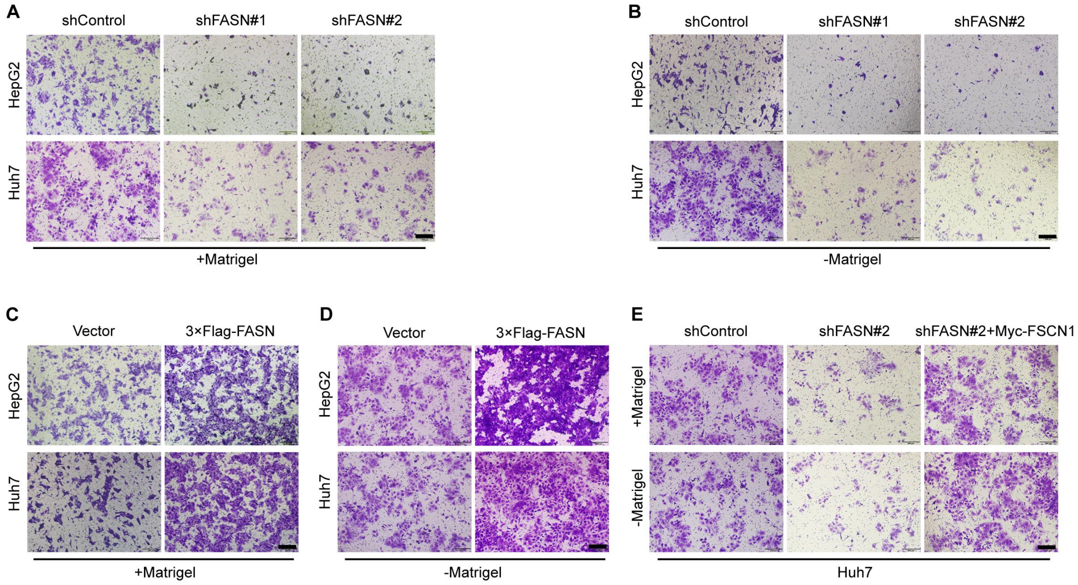

Supplementary Figure 1. (A) Images of HepG2 and Huh7 cells invasion while silencing FASN. Scale bar, 200 μm. **(B)** Images of HepG2 and Huh7 cells migration while silencing FASN. Scale bar, 200 μm. **(C)** Images of HepG2 and Huh7 cells invasion while overexpressing FASN. Scale bar, 200 μm. **(D)** Images of HepG2 and Huh7 cells migration while overexpressing FASN. Scale bar, 200 μm. **(E)** Images of transwell assay of shControl, shFASN#2, and shFASN#2+Myc-FSCN1 groups in Huh7 cells. Scale bar, 200 μm.

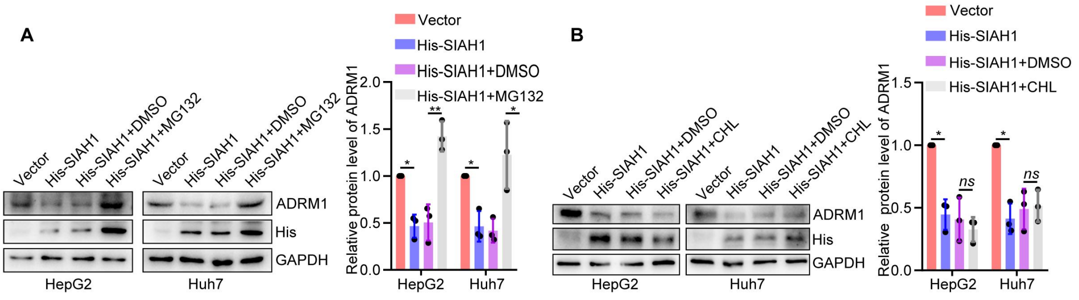

Supplementary Figure 2. (A) Representative bolts and quantification of ADRM1 expression in liver cancer cells transfected with His-SIAH1 plasmid (+MG132). DMSO was used as a solvent control. **(B)** Representative bolts and quantification of ADRM1 expression in liver cancer cells transfected with His-SIAH1 plasmid (+CHL). DMSO was used as a solvent control. * P <0.05, ** P <0.01.

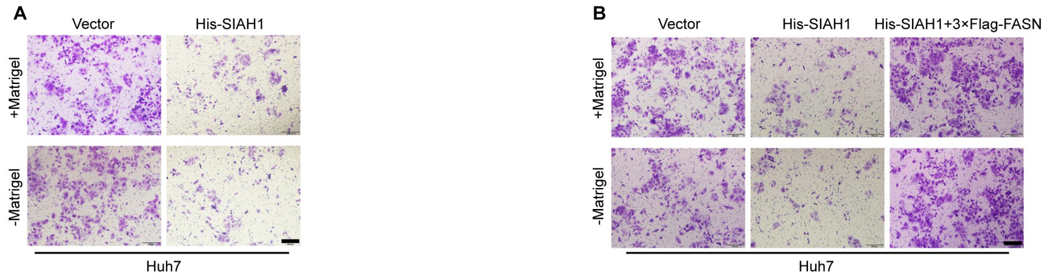

Supplementary Figure 3. (A) Images of Huh7 cells invasion and migration while overexpressing SIAH1. Scale bar, 200 μm. **(B)** Images of transwell assay of Vector, His-SIAH1, and His-SIAH1+3×Flag-FASN groups in Huh7 cells. Scale bar, 200 μm.
