# Supplementary material for: Deficiency of SIAH1 promotes the formation of filopodia by increasing the accumulation of FASN in liver cancer
Source: Cell Death Dis. 2024 Jul 29;15(7):537. doi: 10.1038/s41419-024-06929-7 (PMC11286965; doi:10.1038/s41419-024-06929-7)
Supplement: Supplementary file 2 — Supplementary materials [file 41419_2024_6929_MOESM2_ESM.pdf]

Supplementary Table 1. The antibodies used in this study.

Antibody	Cat No.	Dilution ratio	Manufacturer
FASN	10624-2-AP	1:1000	Proteintech
FSCN1	A1904	1:1000	Abclonal
UCHL5	A7978	1:1000	Abclonal
ADRM1	11468-1-AP	1:2000	Proteintech
SIAH1	13886-1-AP	1:500	Proteintech
HA	51064-2-AP	1:1000	Proteintech
Flag	30503ES80	1:2000	Yeasen
Myc	30601ES60	1:2000	Yeasen
His	66005-1-Ig	1:5000	Proteintech
Ub	10201-2-AP	1:1000	Proteintech
GAPDH	60004-1-Ig	1:4000	Proteintech

Supplementary Table 2. The primer pairs used in this study.

Gene	Type	Sequence (5’-3’)
**shFASN#1**	**FP**	gatccGCATGGAGCGTATCTGTGAGAATTCAAGAGATTCTCACAGATACGCTCCATGTTTTTTg
	**RP**	aattcAAAAAACATGGAGCGTATCTGTGAGAATCTCTTGAATTCTCACAGATACGCTCCATGCg
**shFASN#2**	**FP**	gatccGCCTACTGGATGCGTTCTTCAATTCAAGAGATTGAAGAACGCATCCAGTAGGTTTTTTg
	**RP**	aattcAAAAAACCTACTGGATGCGTTCTTCAATCTCTTGAATTGAAGAACGCATCCAGTAGGCg
**shFASN#3**	**FP**	gatccGCTACGACTACGGCCCTCATTTTCAAGAGAAATGAGGGCCGTAGTCGTAGCTTTTTT g
	**RP**	aattcAAAAAAGCTACGACTACGGCCCTCATTTCTCTTGAAAATGAGGGCCGTAGTCGTAGCg
**shUCHL5**	**FP**	gatccGCAGAAGATAGCAGAGTTACATTCAAGAGATGTAACTCTGCTATCTTCTGCTTTTTTg
	**RP**	aattcAAAAAAGCAGAAGATAGCAGAGTTACATCTCTTGAATGTAACTCTGCTATCTTCTGCg
**shSIAH1#1**	**FP**	GGATCCGGAAAGGCTACTCCACCTTCTTTCAAGAGAAGAAGGTGGAGTAGCCTTTCCTTTTTTG
	**RP**	AATTCAAAAAAGGAAAGGCTACTCCACCTTCTTCTCTTGAAAGAAGGTGGAGTAGCCTTTCCG
**shSIAH1#2**	**FP**	GGATCCGGAAAGGCTACTCCACCTTCTTTCAAGAGAAGAAGGTGGAGTAGCCTTTCCTTTTTTG
	**RP**	AATTCAAAAAAGGAAAGGCTACTCCACCTTCTTCTCTTGAAAGAAGGTGGAGTAGCCTTTCCG

background of the liver cancer specimen

FASN low								
Name	ID	Gender	Tumor size, cm	Number of tumor nodules	MVI	T	N	M
Pan Peiyin	2030791	Male	2	1	0	1	0	0
Zhu Shule	2053213	Male	2	1	0	1	0	0
Li Hongyan	2072817	Male	2	1	0	1	0	0
Xia Fengying	2061879	Female	2	1	0	1	0	0
Wang Mingrong	2048712	Female	2.4	1	0	1	0	0
Sun Jiming	2027653	Male	2.5	1	0	1	0	0
Li Ronghua	2058393	Female	2.5	1	0	1	0	0
Gong Mingjiang	2207505	Male	3	1	0	1	0	0
Xin Shuhua	2046718	Female	3.5	1	0	1	0	0
Song Juxing	2141635	Male	4.5	1	0	1	0	0
Feng Zunyi	2113974	Male	3	2	0	1	0	0
Wu Guangdong	1967095	Male	2.5	1	0	2	0	0
Zhao Houmin	3186685	Male	3.5	1	0	2	0	0
Ji Chuanjiang	1891886	Male	5.5	1	0	2	0	0
Lu Shibao	1895775	Male	5.5	1	0	2	0	0
Guo Huabao	1966399	Male	6.5	1	0	2	0	0
Qi Bangjun	3066985	Male	10.7	1	0	2	0	0
Wei Chuanhong	1893775	Male	2	2	0	2	0	0
Wang Hongyuan	2004827	Male	5	1	0	3	0	0
Guo Aimin	3357063	Male	5.3	1	0	3	0	0
Liu Junmin	2080773	Male	16	1	0	3	0	0
Li Xiaohu	3237971	Male	3	2	0	4	0	0
Wang Jinchao	3052627	Male	2.4	1	0	2	1	0
Wang Jianfei	3029423	Male	3	1	1	2	0	0
Zhu Cuiping	3028931	Female	2	2	1	2	0	0
Li Dongjuan	3186055	Female	3.8	1	1	3	0	0
Wang Qingyang	3049034	Female	5.5	1	1	3	0	0
Meng Xiangji	3156675	Male	8	1	1	3	0	0
Zhou Aiyun	2024385	Female	1.5	1	1	4	0	0
XU Shanwei	2128346	Male	5.7	1	1	4	0	0
Zhou Fengping	2055860	Female	8	1	1	4	0	0
Liu Tidong	1927944	Male	6.5	2	1	4	0	0
Hou Jianming	1985443	Male	4	1	2	3	0	0
Zhu Chengxia	1983569	Female	13	1	2	3	0	0
Lu Deqian	3497513	Male	5	>3	2	3	0	0
Liu Hefu	3253249	Male	6	1	2	4	0	0
Yang Zhaohua	3253176	Male	13	2	2	4	0	0
Jiang Renshan	1998794	Male	1.5	3	2	4	0	0

FASN high								
Name	ID	Gender	Tumor size, cm	Number of tumor nodules	MVI	T	N	M
Tong Meiyan	3136318	Female	1.3	1	0	1	0	0
Zhu Huaixia	2082035	Female	2	1	0	1	0	0
Yin Zilan	3109951	Female	2.5	1	0	1	0	0
Du Wenling	3417668	Female	2.6	1	0	1	0	0
Wu Yan	3421550	Female	5.8	1	0	1	0	0
Liu Maojun	1973964	Male	6.5	1	0	1	0	0
Zhang Huating	3049934	Male	7	1	0	1	0	0
Sun Zhongmin	1878663	Male	3	1	0	2	0	0
Liu Yonghuai	3353341	Male	20	1	0	2	0	0
Zhang Guiping	3049979	Female	3.5	2	0	2	0	0
Zhu Liyi	2022590	Male	4.3	2	0	2	0	0
Liu Meixia	1951276	Female	11	2	0	2	0	0
Li Zhenwu	1989442	Male	2.2	3	0	2	0	0
Zhang Gongqian	2124390	Male	6	1	0	3	0	0
Han Hongyang	1906006	Male	6.5	1	0	3	0	0
Zhang Bentian	3392080	Male	10	1	0	3	0	0
Li Jingping	1895007	Male	2.7	2	0	3	0	0
Xu Fanghe	3042222	Male	2.7	2	0	3	0	0
Chen Shiwu	2013215	Male	18	1	0	4	0	0
Yue Chongyuan	3335932	Male	6.5	1	1	3	0	0
Yan Xizhong	3358126	Male	9	1	1	3	0	0
Ren Zhongli	3153852	Male	15	1	1	3	0	0
Wang Fasheng	3204415	Male	15	1	1	3	0	0
Bao Qipei	3352873	Male	6.5	2	1	3	0	0
Liu Bangchao	3033505	Male	10	2	1	3	0	0
Zhao Qingyu	3087418	Male	9	3	1	3	0	0
Sun Yanmei	2043592	Female	5.8	1	1	4	0	0
Zhang Zhigao	3544744	Male	10	1	1	4	0	0
Pei Hongyi	3441663	Male	13	1	1	4	0	0
Wang Xianjun	3125001	Male	6.5	1	1	2	1	0
Mao Lifa	1991931	Male	7.5	1	2	3	0	0
Li Jiaping	2074232	Male	6.5	1	2	4	0	0

Days of animal survival after tail vein injection

shControl	shFASN#1+2
51	77
55	91
73	103
83	108
87	109
87	120
88	120
88	128
95	128
100	132
102	132
102	146
104	146
106	146
106	146
108	146
111	152
124	152
132	176
132	178
135	178
142	182
142	182
145	182
151	190

Original Figures

Figure 1

Figure 1E

FASN

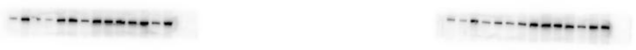

GAPDH

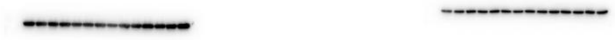

Figure 1F

FASN/HepG2 and Huh7

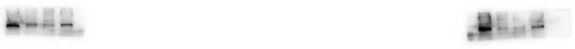

GAPDH/HepG2 and Huh7

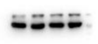

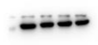

Figure 2

Figure 2C

FSCN1/HepG2 and Huh7

FASN/HepG2 and Huh7

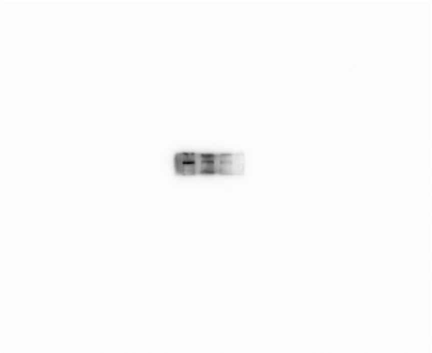

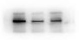

GAPDH/HHepG2 and Huh7

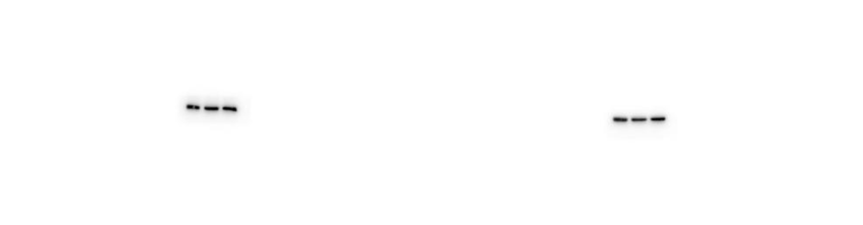

Figure 2D

FSCN1/HepG2 and Huh7

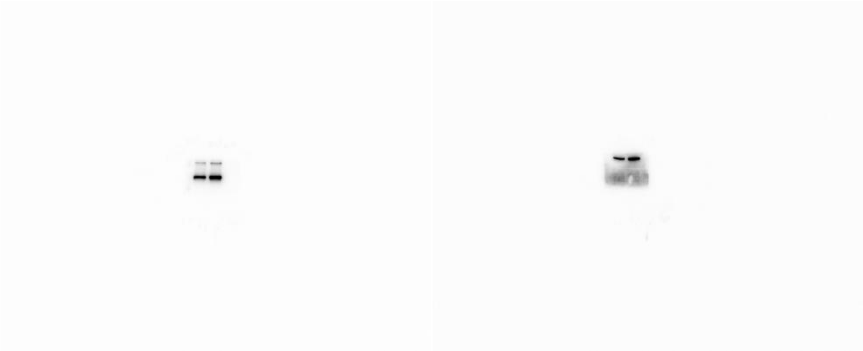

3*Flag-FASN/HepG2 and Huh7

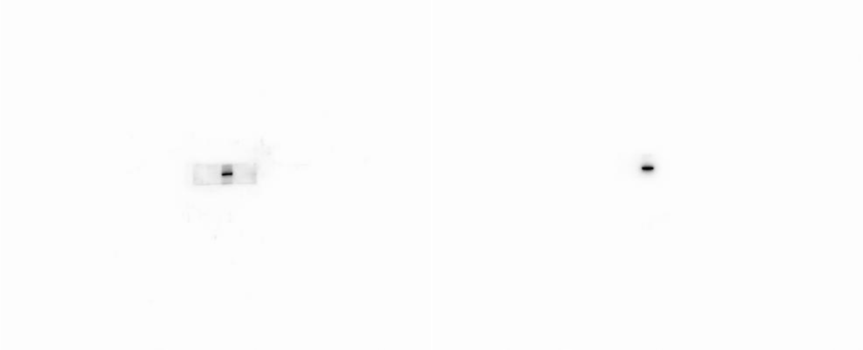

GAPDH/HepG2 and Huh7

Figure 2E

Silencing of FASN

CDC42/HepG2 and Huh7

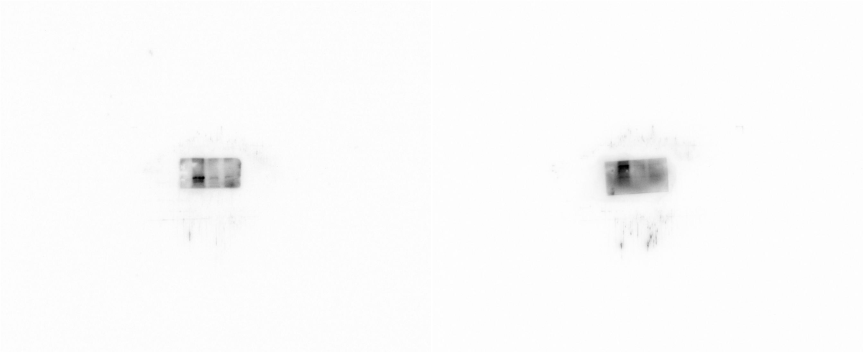

RAC1/HepG2 and Huh7

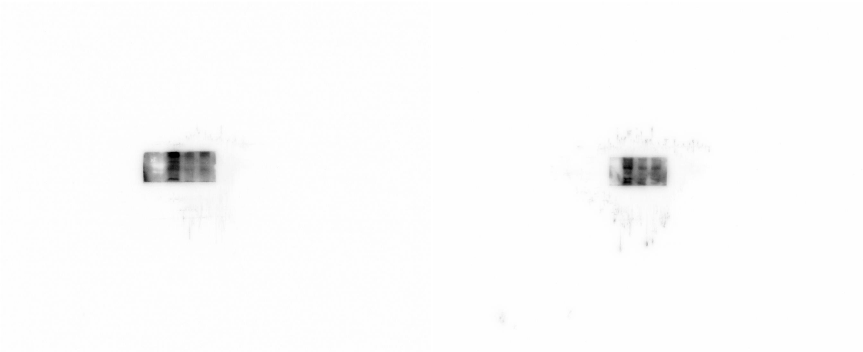

RHOA/HepG2 and Huh7

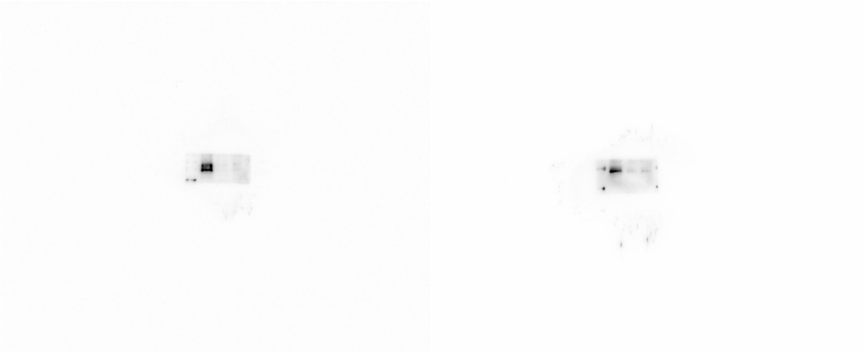

FASN/HepG2 and Huh7

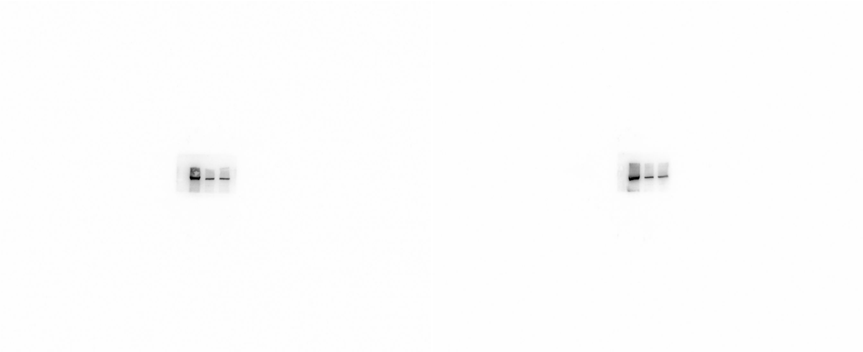

GAPDH/HepG2 and Huh7

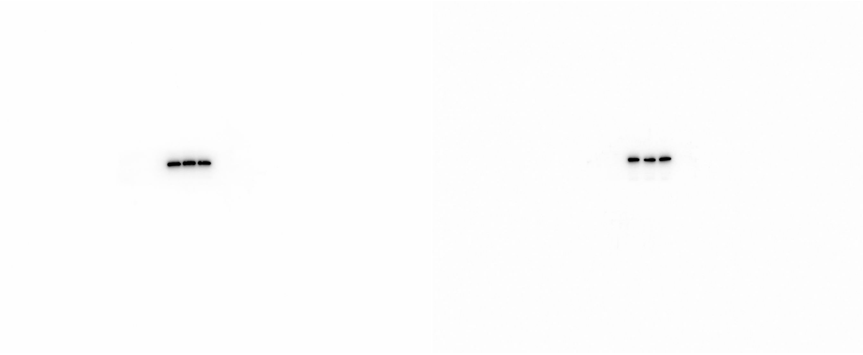

Overexpressing of FASN

CDC42/HepG2 and Huh7

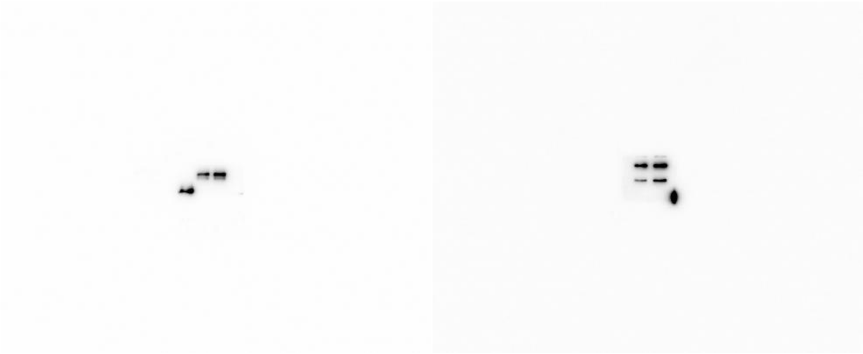

RAC1/HepG2 and Huh7

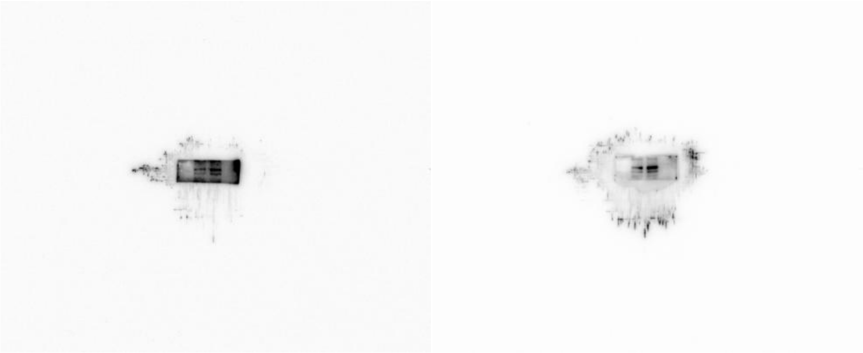

RHOA/HepG2 and Huh7

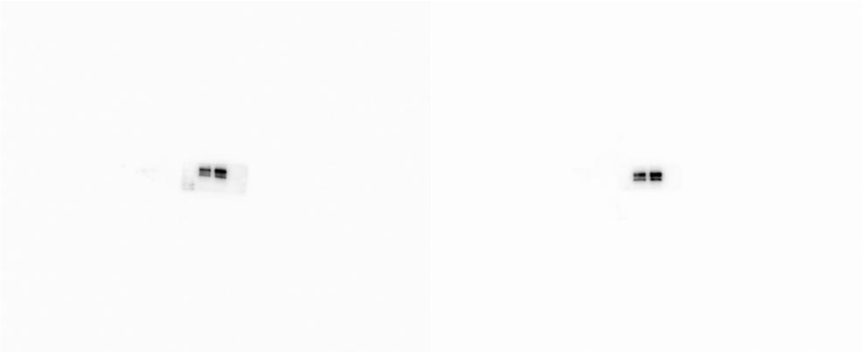

3*Flag-FASN/HepG2 and Huh7

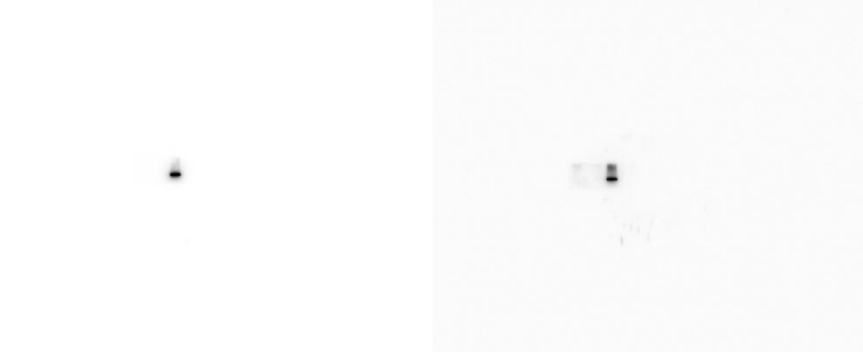

GAPDH/HepG2 and Huh7

Figure 3

Figure 3C

E-cadherin/HepG2 and Huh7

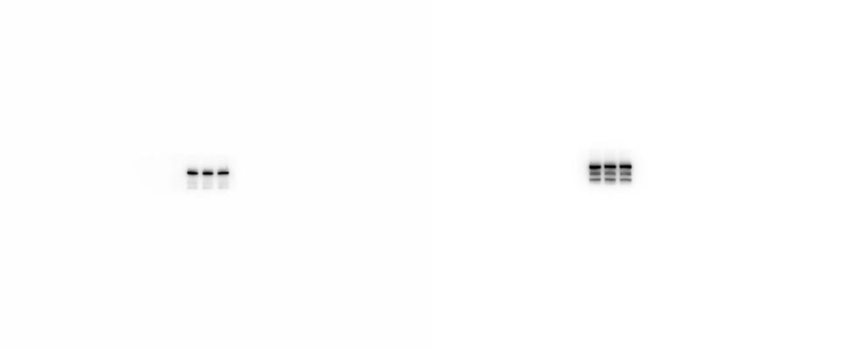

N-cadherin/HepG2 and Huh7

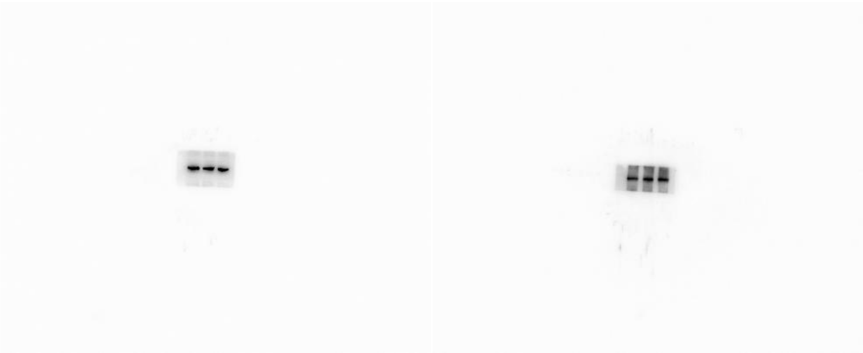

MMP2/HepG2 and Huh7

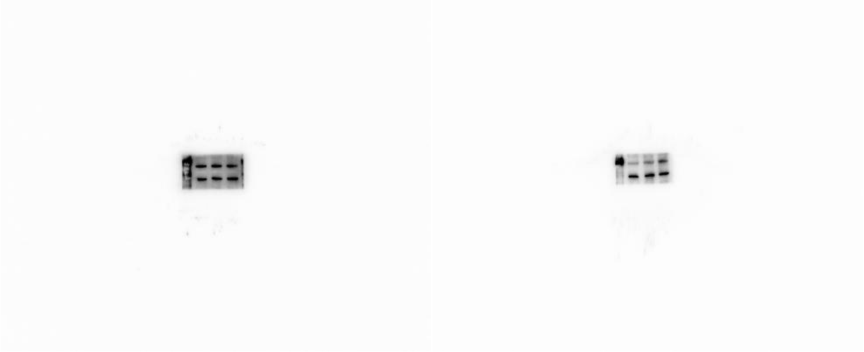

MMP9/HepG2 and Huh7

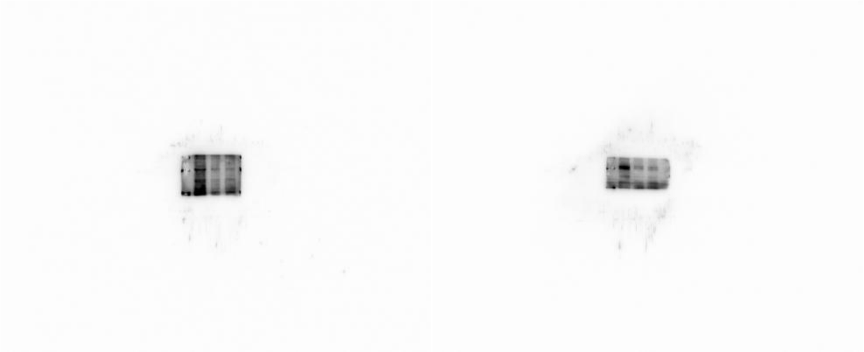

FASN/HepG2 and Huh7

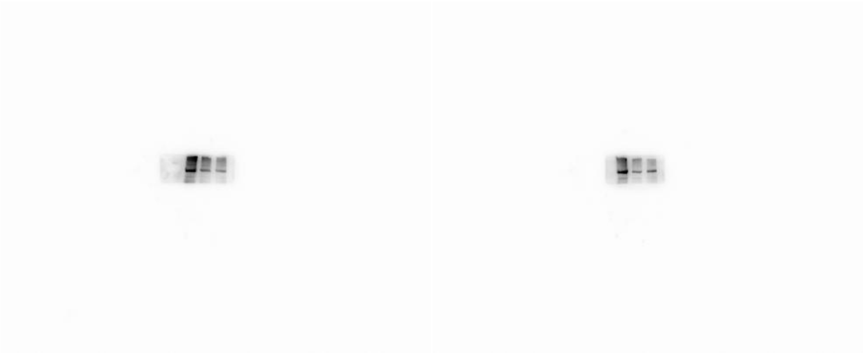

GAPDH/HepG2 and Huh7

Figure 3C

E-cadherin/HepG2 and Huh7

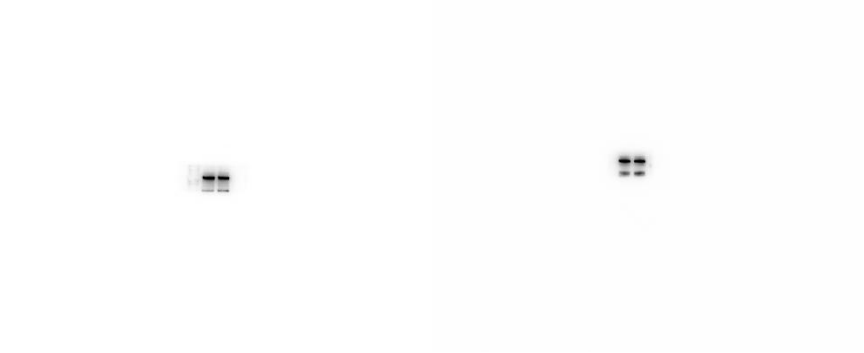

N-cadherin/HepG2 and Huh7

MMP2/HepG2 and Huh7

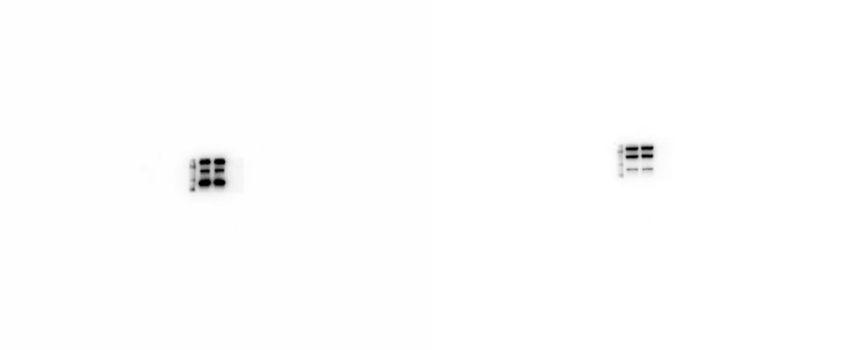

MMP9/HepG2 and Huh7

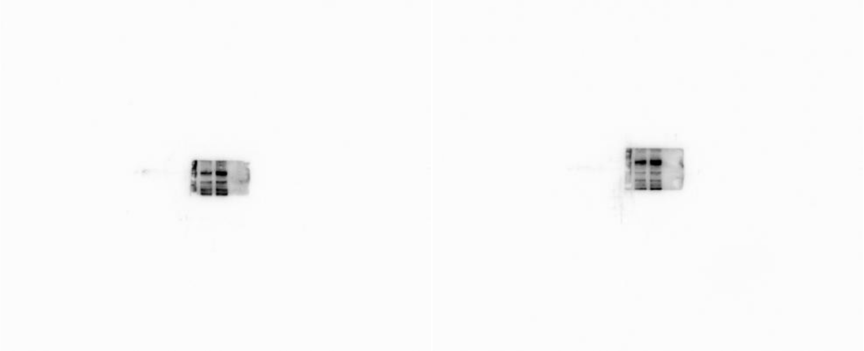

3*Flag-FASN/HepG2 and Huh7

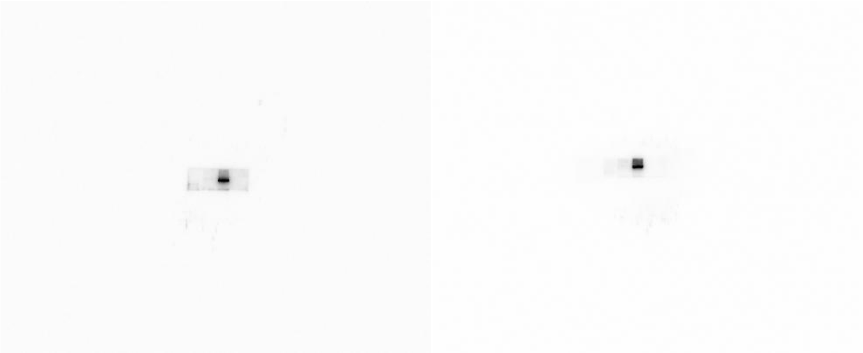

GAPDH/HepG2 and Huh7

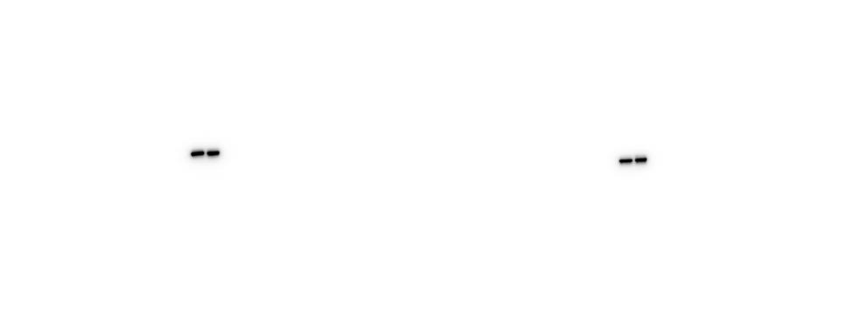

Figure 3E

FSCN1

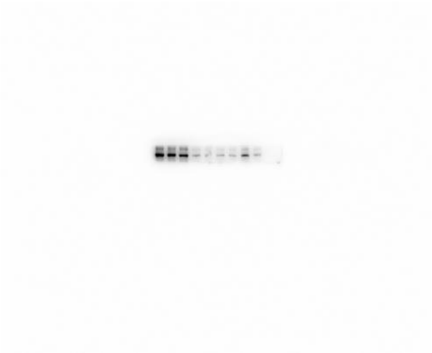

FASN

GAPDH

Figure 3H

FASN

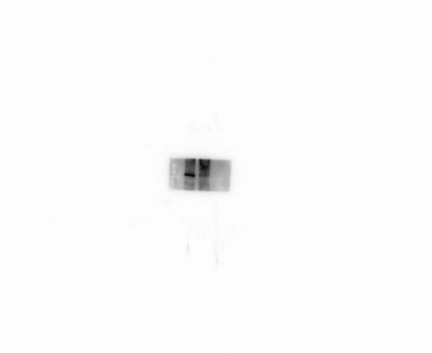

GAPDH

Figure 4

Figure 4A

CHL
FASN/HepG2 and Huh7

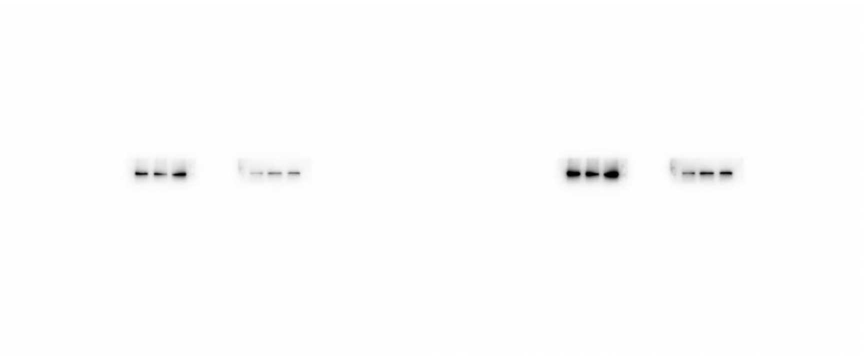

GAPDH/HepG2 and Huh7

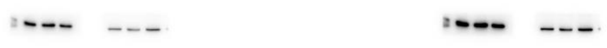

MG132

FASN/HepG2 and Huh7

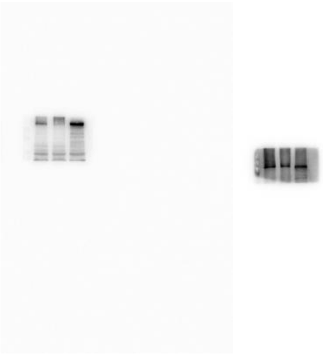

GAPDH/HepG2 and Huh7

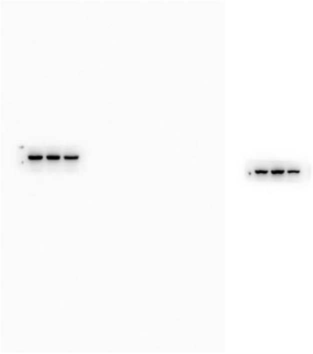

Figure 4B

Ub/HepG2 and Huh7

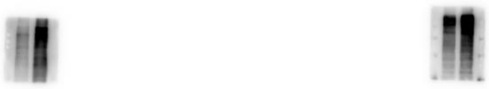

FASN/HepG2 and Huh7

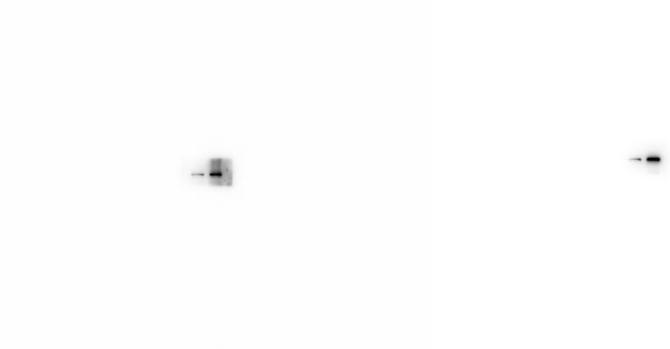

GAPDH/HepG2 and Huh7

Figure 4C

FASN/HepG2 and Huh7

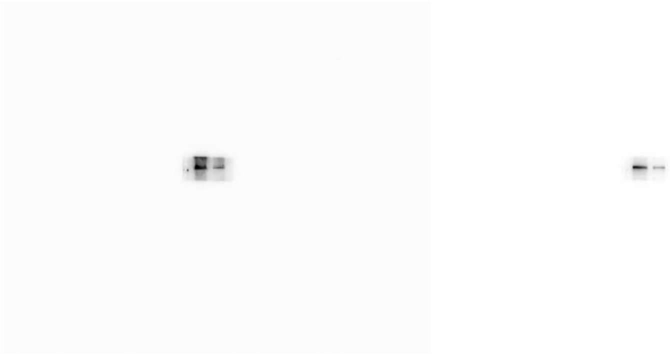

UCHL5/HepG2 and Huh7

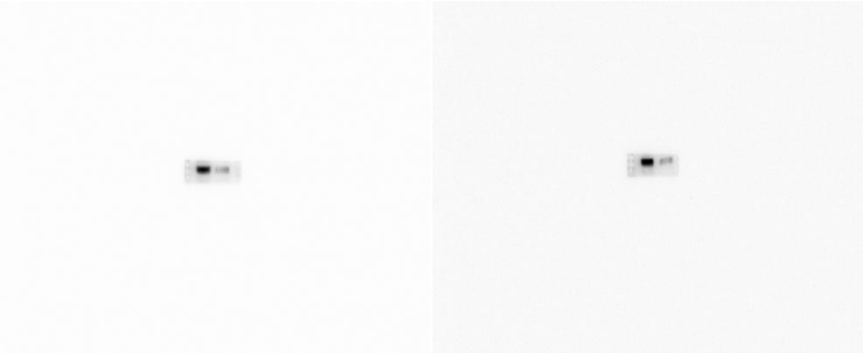

GAPDH/HepG2 and Huh7

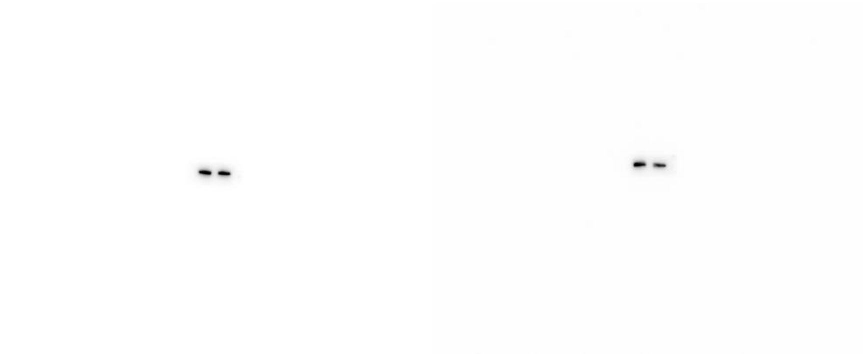

Figure 4D

FASN/HepG2 and Huh7

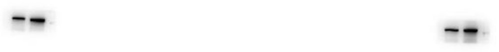

UCHL5/HepG2 and Huh7

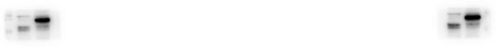

GAPDH/HepG2 and Huh7

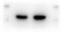

Figure 4E
UCHL5/HepG2

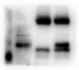

FASN/HepG2

UCHL5/Huh7

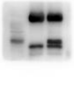

FASN/Huh7

Figure 4F

Ub/HepG2 and Huh7

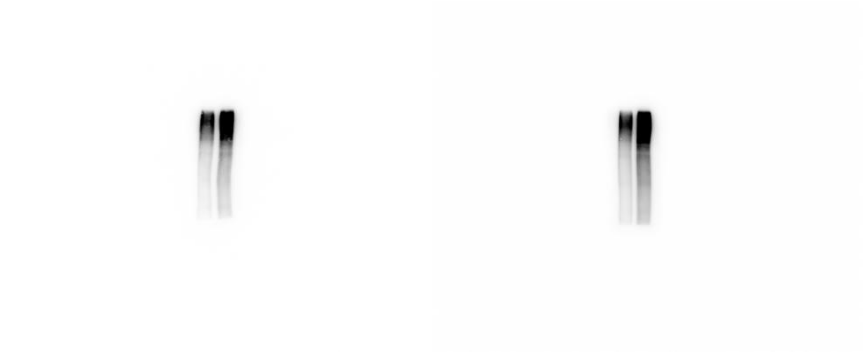

FASN/HepG2 and Huh7

UCHL5/HepG2 and Huh7

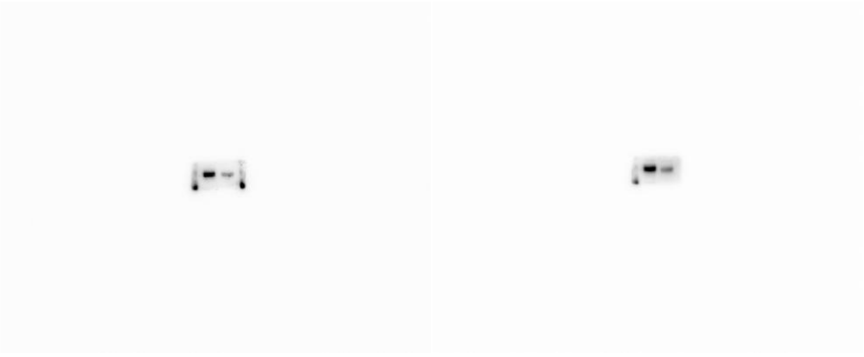

GAPDH/HepG2 and Huh7

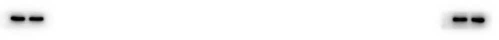

Figure 4G

Ub/HepG2 and Huh7

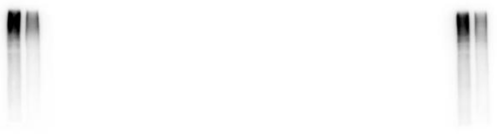

FASN/HepG2 and Huh7

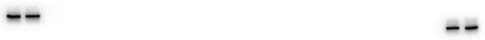

UCHL5/HepG2 and Huh7

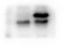

GAPDH/HepG2 and Huh7

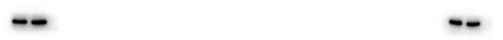

Figure 4H

HA/HepG2 and Huh7

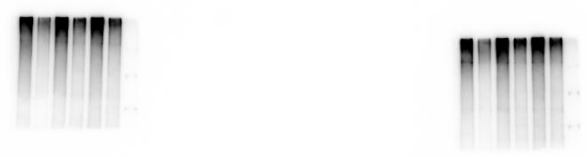

FASN/HepG2 and Huh7

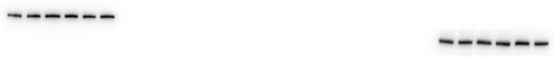

UCHL5/HepG2 and Huh7

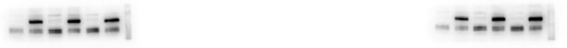

GAPDH/HepG2 and Huh7

Figure 4I

FASN/HepG2 and Huh7

UCHL5/HepG2 and Huh7

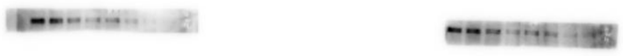

GAPDH/HepG2 and Huh7

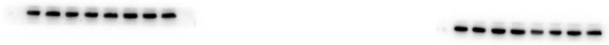

Figure 4L

UCHL5

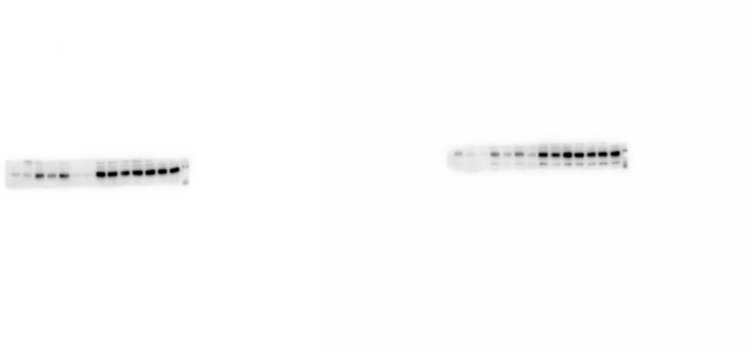

GAPDH

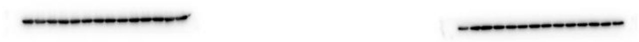

Figure 5

Figure 5A

FSCN1/HepG2 and Huh7

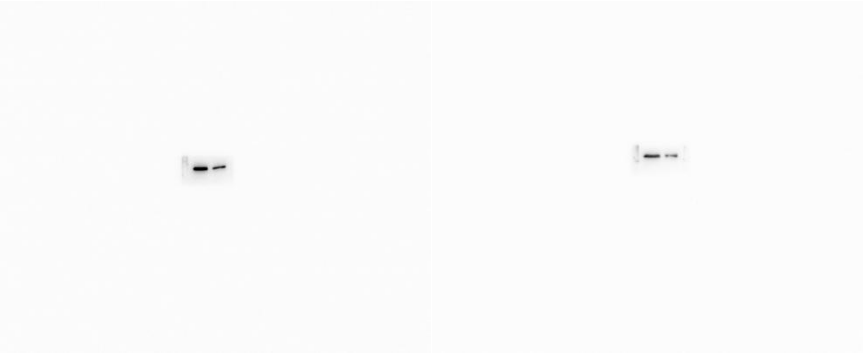

GAPDH/HepG2 and Huh7

Figure 5B

FSCN1/HepG2 and Huh7

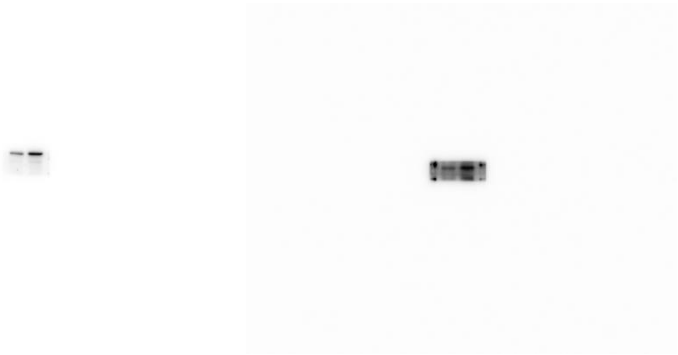

GAPDH/HepG2 and Huh7

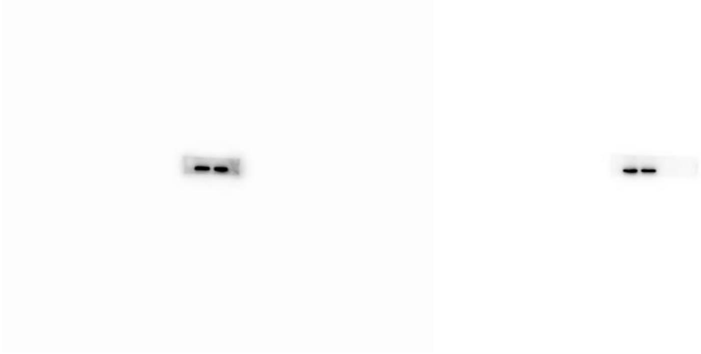

Figure 5D

FSCN1/HepG2 and Huh7

3×Flag-FASN/HepG2 and Huh7

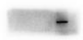

UCHL5/HepG2 and Huh7

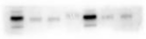

GAPDH/HepG2 and Huh7

Figure 6

Figure 6A

FSCN1/HepG2 and Huh7

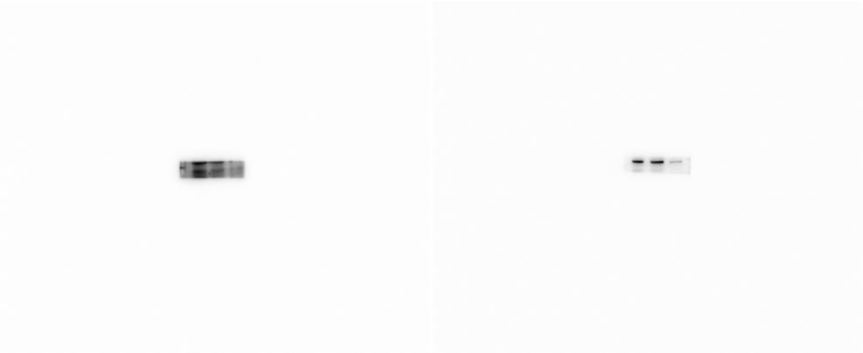

FASN/HepG2 and Huh7

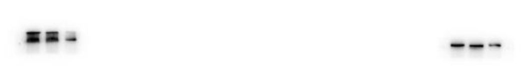

UCHL5/HepG2 and Huh7

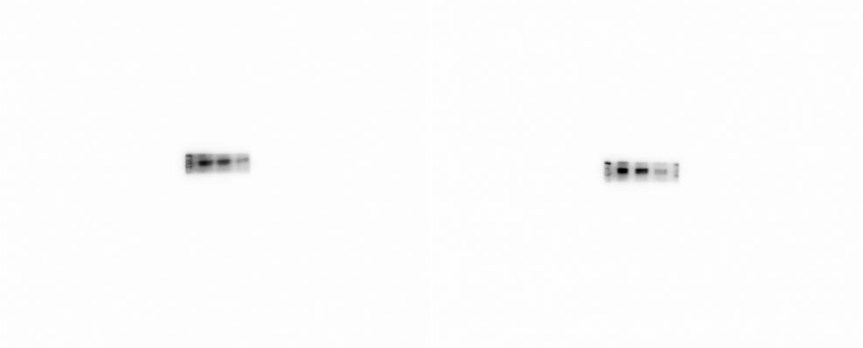

ADRM1/HepG2 and Huh7

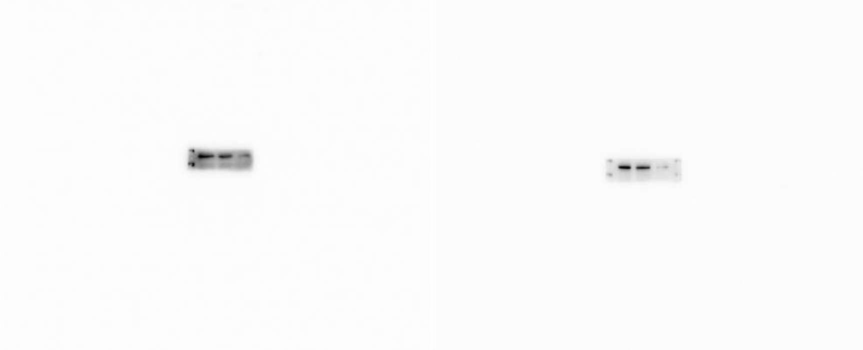

GAPDH/HepG2 and Huh7

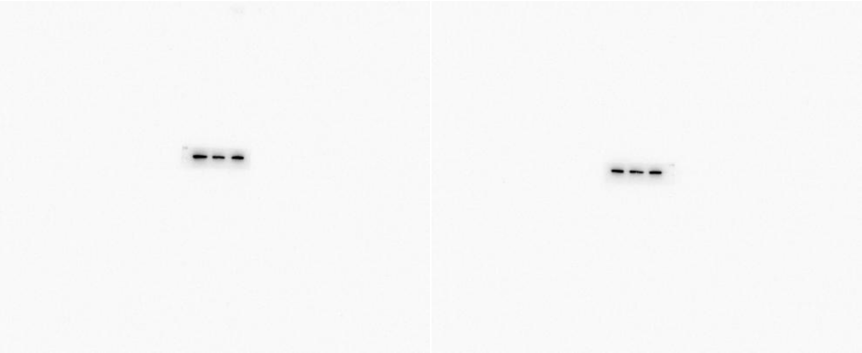

Figure 6B

FSCN1/HepG2 and Huh7

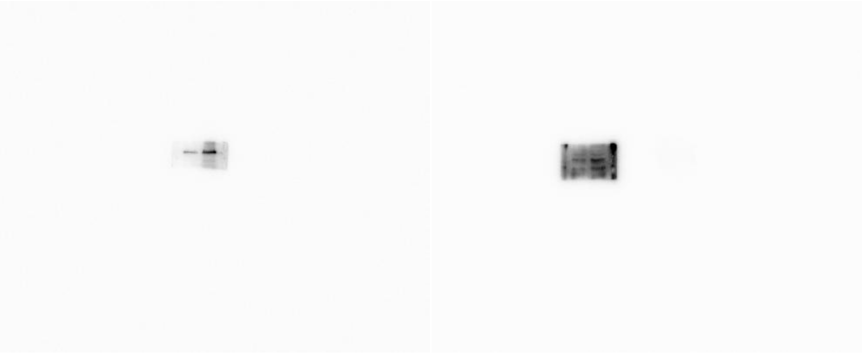

FASN/HepG2 and Huh7

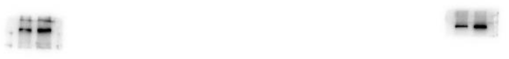

UCHL5/HepG2 and Huh7

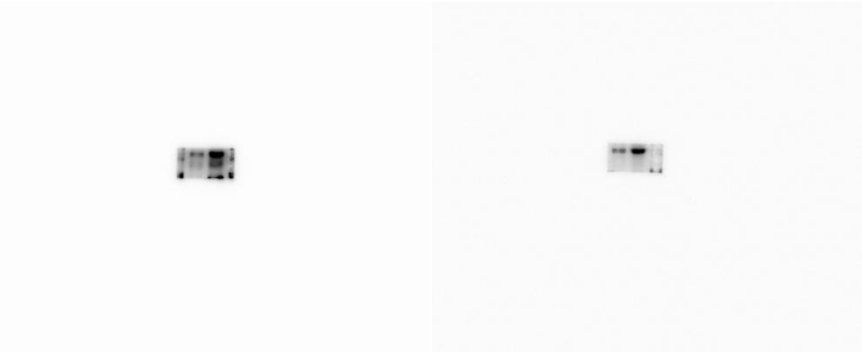

Myc-ADRM1/HepG2 and Huh7

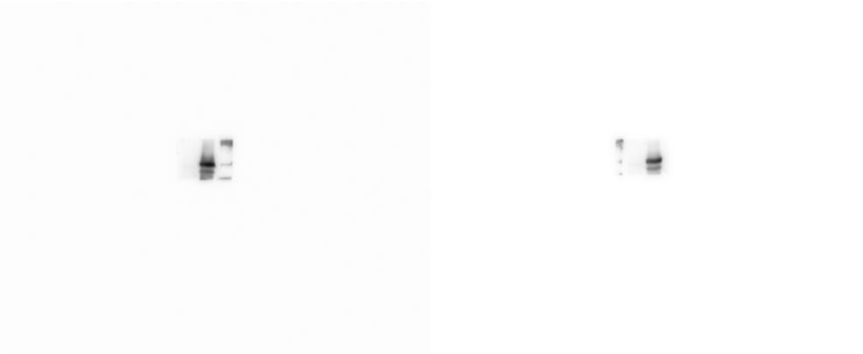

GAPDH/HepG2 and Huh7

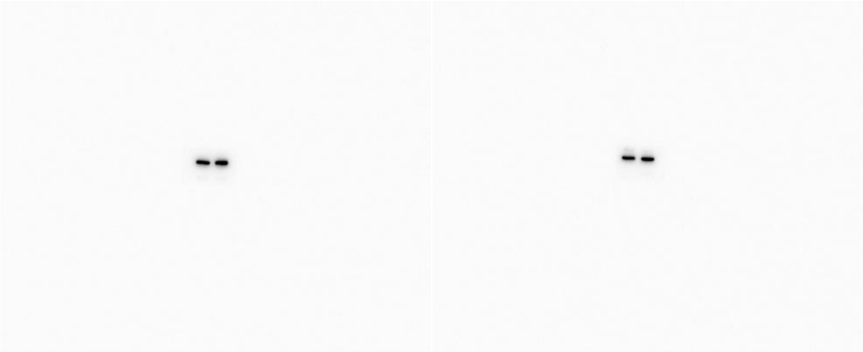

Figure 6C

ADRM1/HepG2 and Huh7

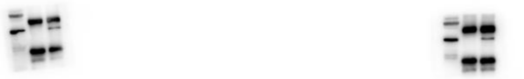

FASN/HepG2 and Huh7

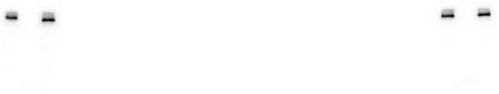

UCHL5/HepG2 and Huh7

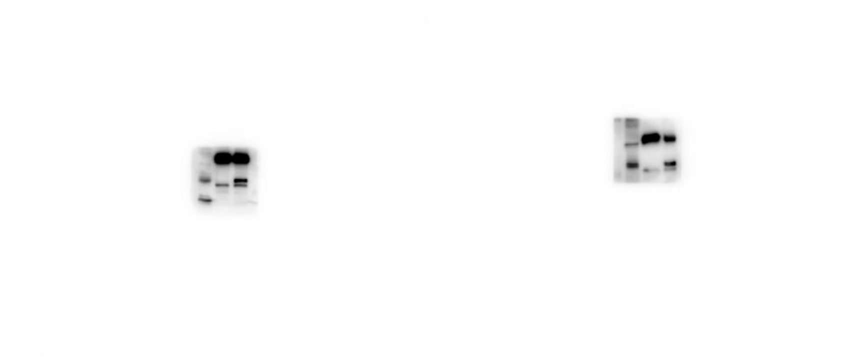

ADRM1/HepG2 and Huh7

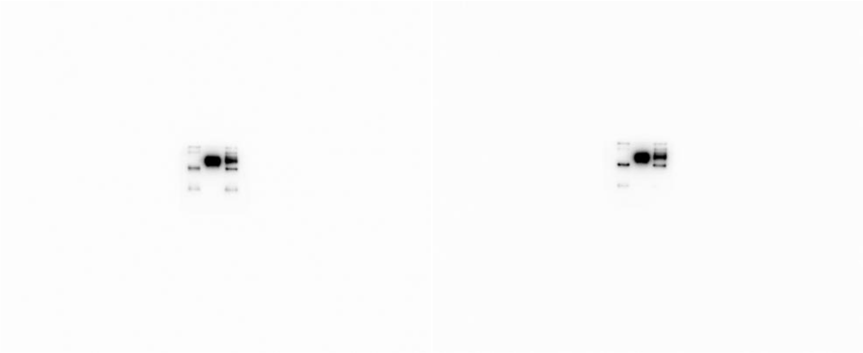

Figure 6D

Ub/HepG2 and Huh7

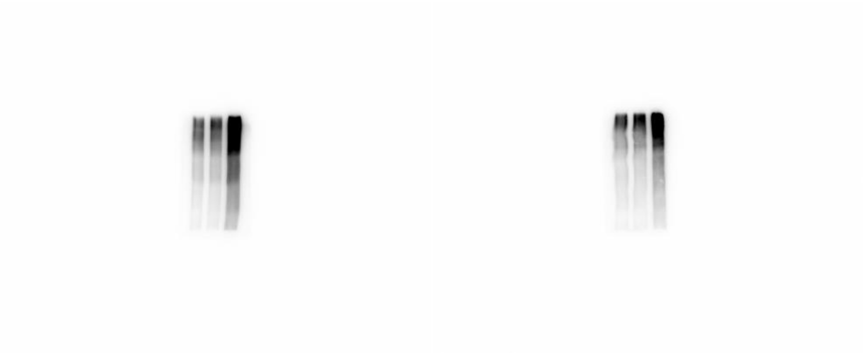

FASN/HepG2 and Huh7

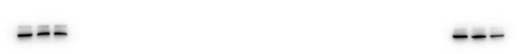

ADRM1/HepG2 and Huh7

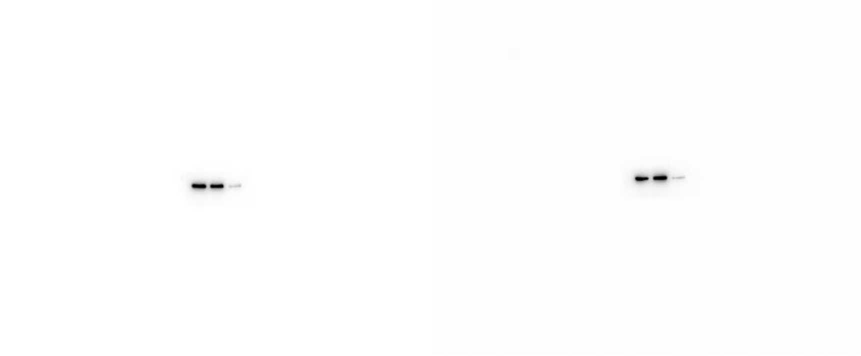

GAPDH/HepG2 and Huh7

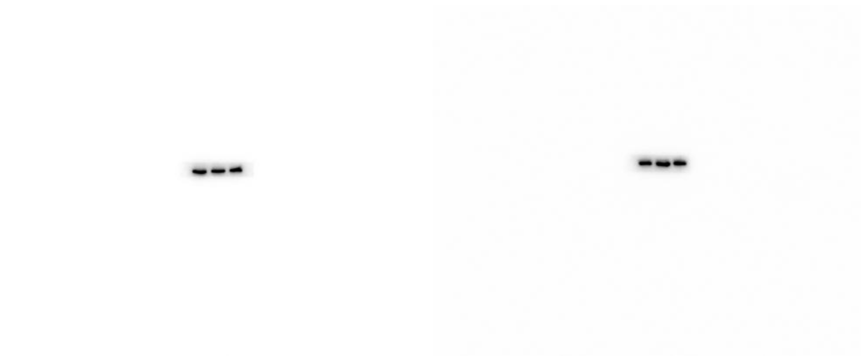

Figure 6E

Ub/HepG2 and Huh7

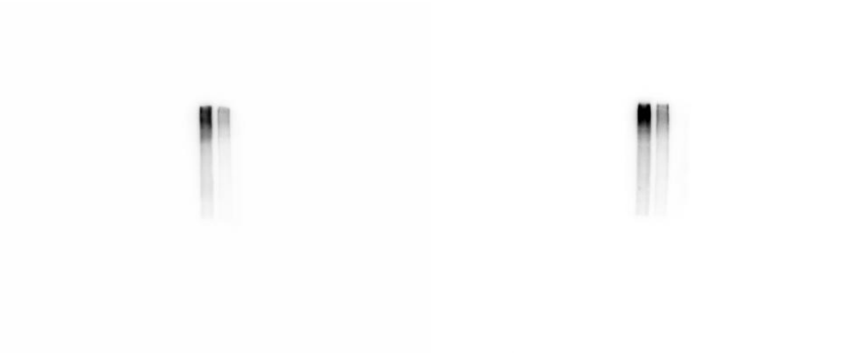

FASN/HepG2 and Huh7

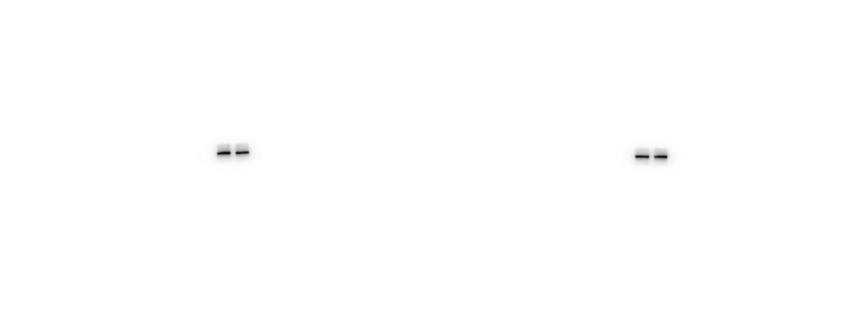

Myc-ADRM1/HepG2 and Huh7

GAPDH/HepG2 and Huh7

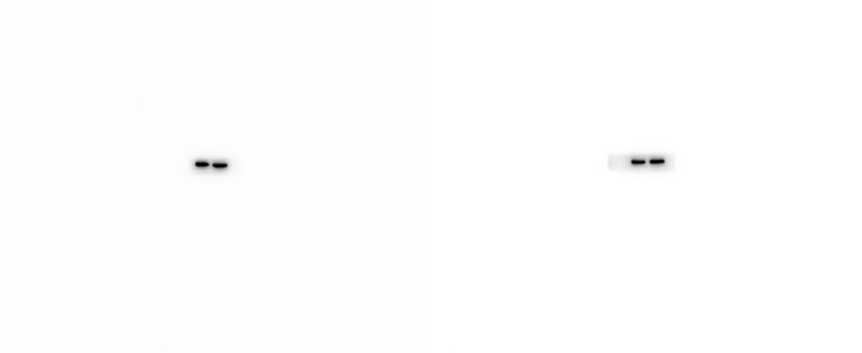

Figure 6I

ADRM1

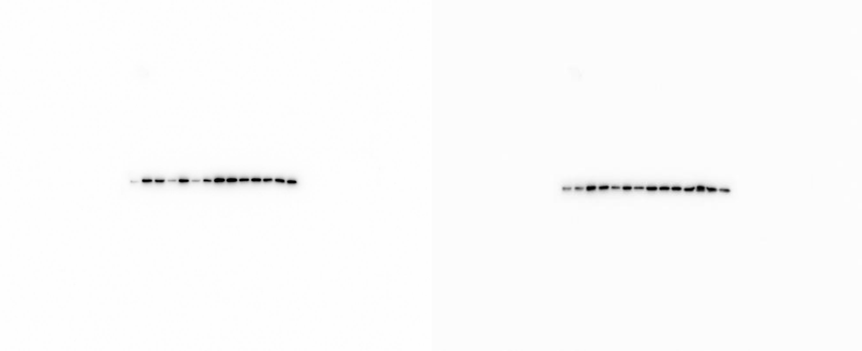

GAPDH

Figure 7

Figure 7B

UCHL5/HepG2 and Huh7

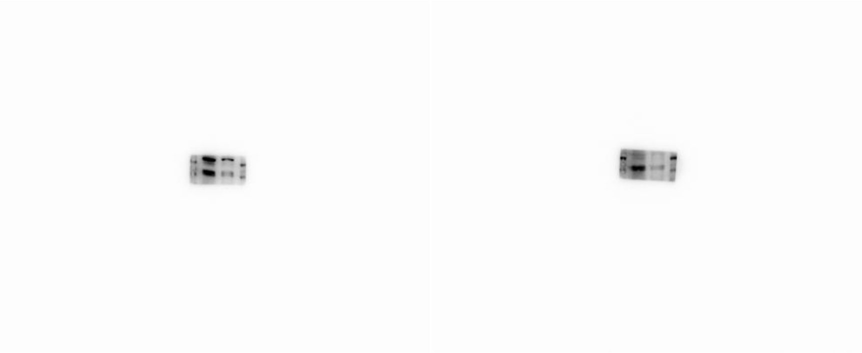

ADRM1/HepG2 and Huh7

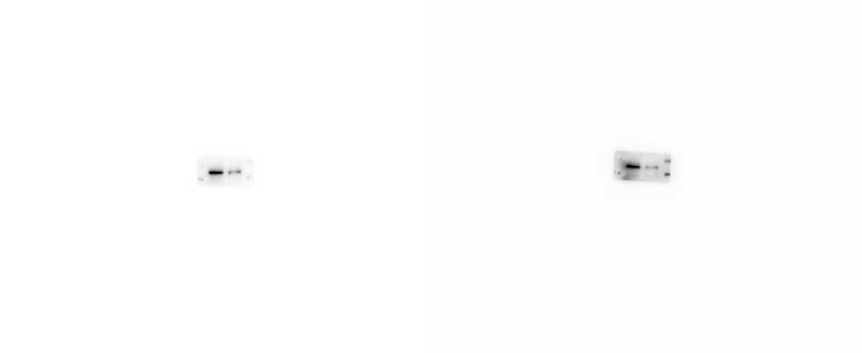

His-SIAH1/HepG2 and Huh7

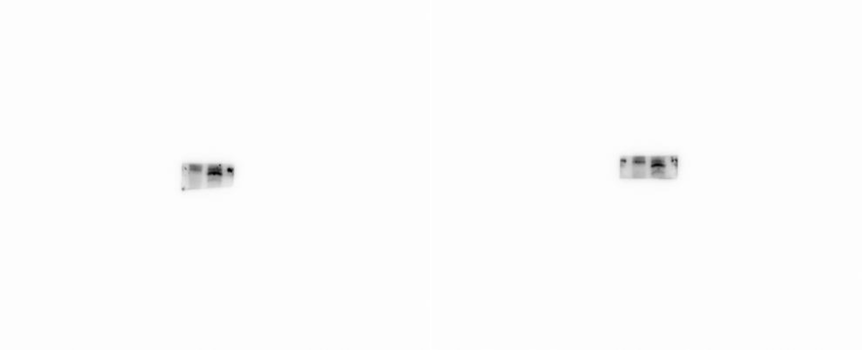

GAPDH/HepG2 and Huh7

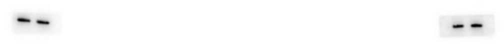

Figure 7C

UCHL5/HepG2 and Huh7

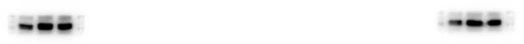

ADRM1/HepG2 and Huh7

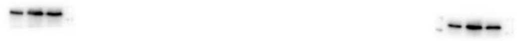

SIAH1/HepG2 and Huh7

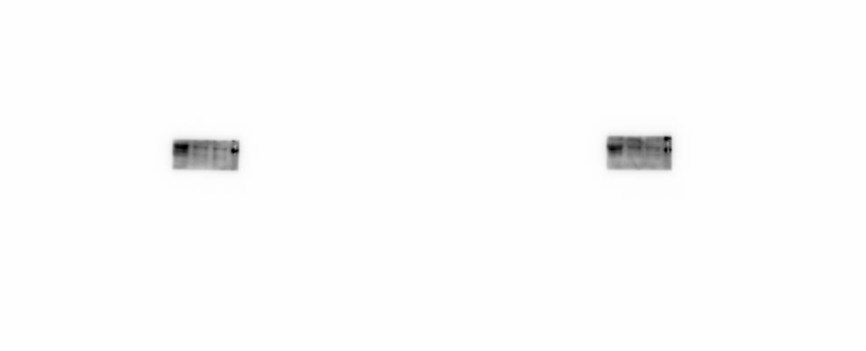

GAPDH/HepG2 and Huh7

Figure 7D

ADRM1/HepG2 and Huh7

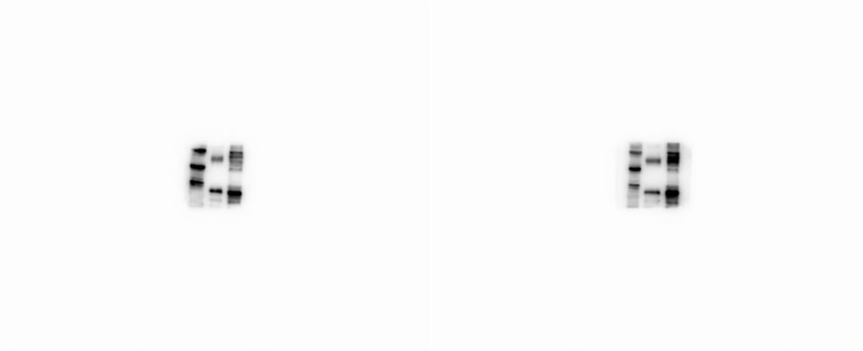

SIAH1/HepG2 and Huh7

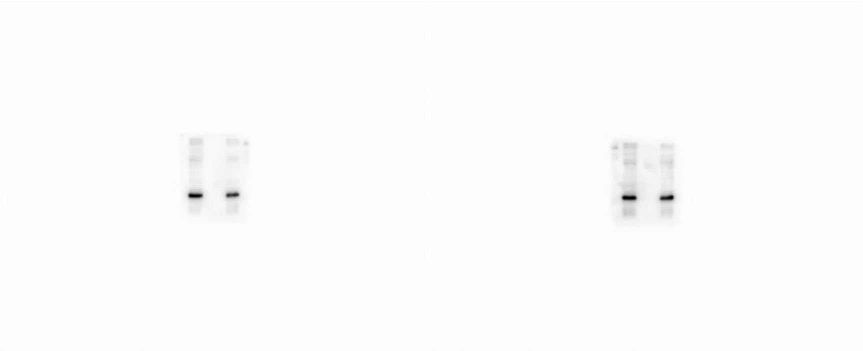

UCHL5/HepG2 and Huh7

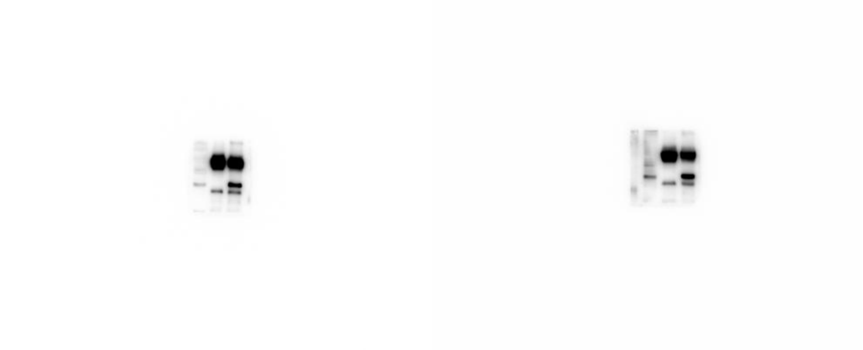

SIAH1/HepG2 and Huh7

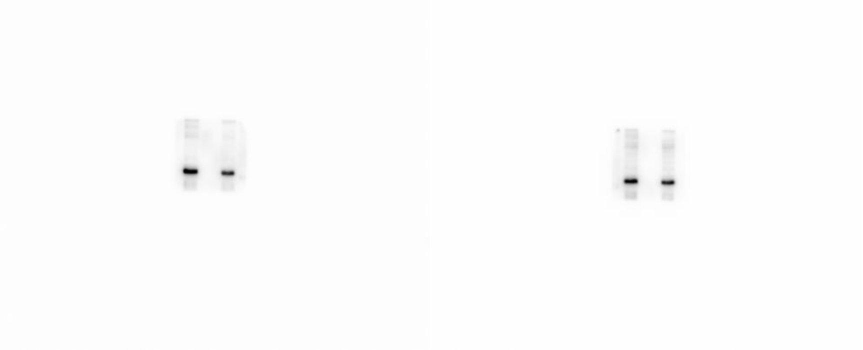

Figure 7E

ADRM1/HepG2 and Huh7

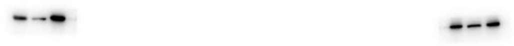

His-SIAH1/HepG2 and Huh7

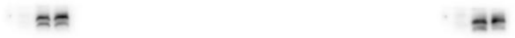

GAPDH/HepG2 and Huh7

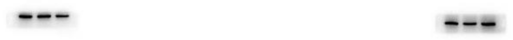

Figure 7F

Ub/HepG2 and Huh7

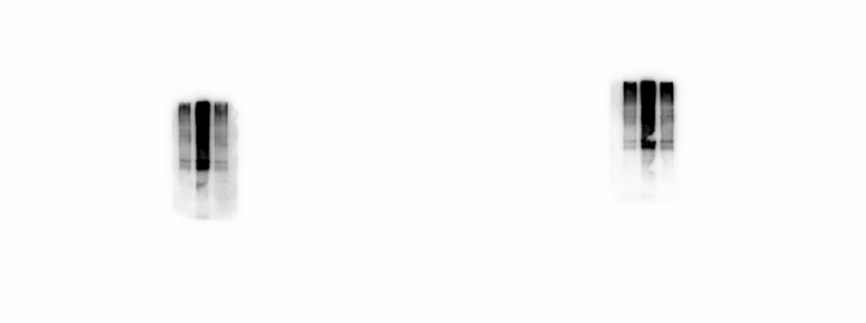

ADRM1/HepG2 and Huh7

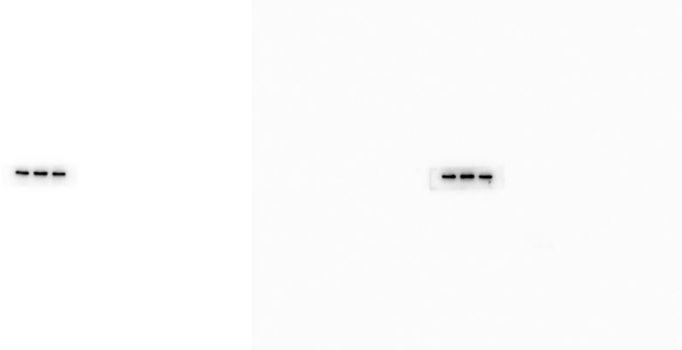

His-SIAH1/HepG2 and Huh7

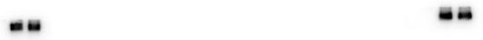

GAPDH/HepG2 and Huh7

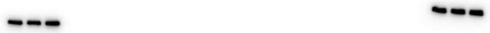

Figure 7G

ADRM1

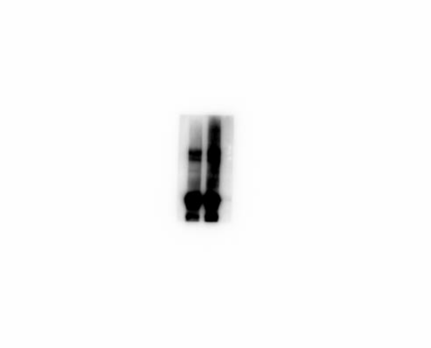

Figure 7H

HA/HepG2 and Huh7

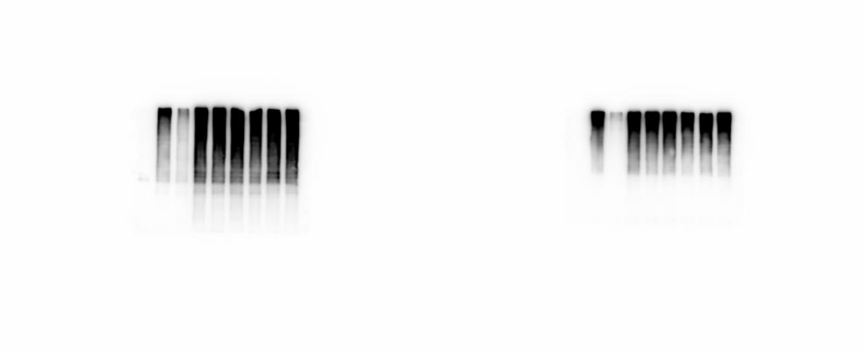

ADRM1/HepG2 and Huh7

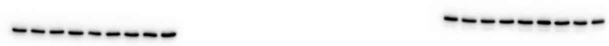

His-SIAH1/HepG2 and Huh7

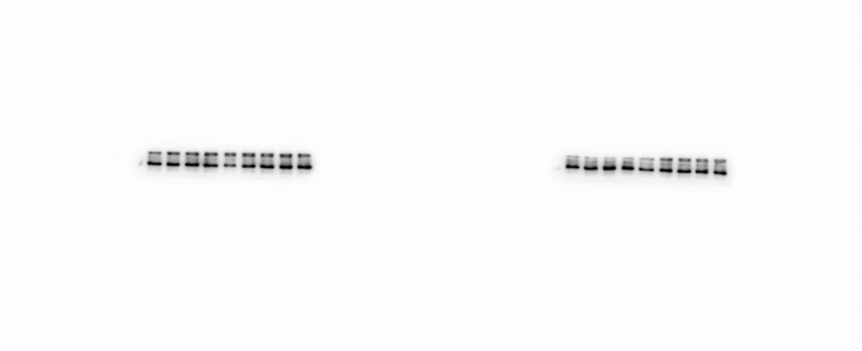

GAPDH/HepG2 and Huh7

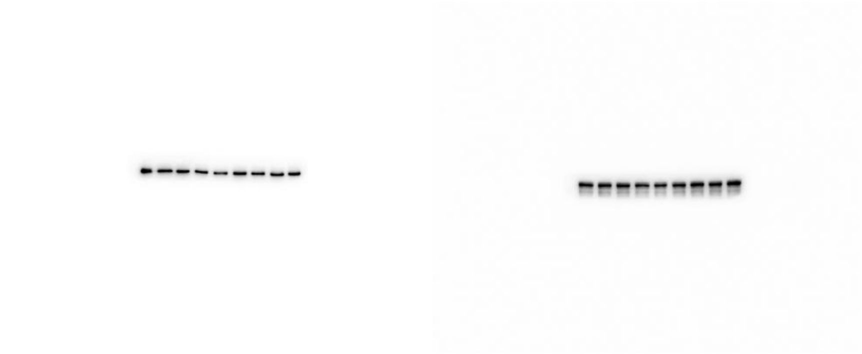

Figure 7I

ADRM1/HepG2 and Huh7

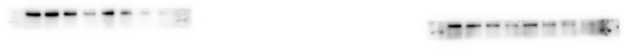

His-SIAH1/HepG2 and Huh7

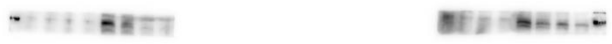

GAPDH/HepG2 and Huh7

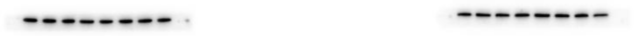

Figure 7J

SIAH1/HepG2 and Huh7

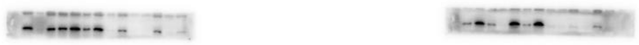

GAPDH/HepG2 and Huh7

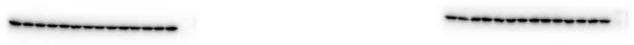

Figure 8

Figure 8A

FASN/HepG2 and Huh7

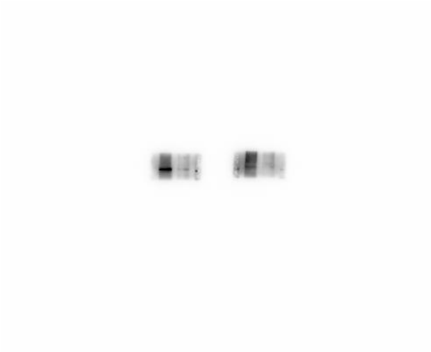

His-SIAH1/HepG2 and Huh7

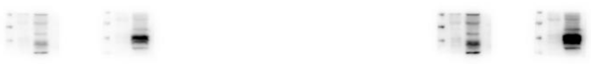

GAPDH/HepG2 and Huh7

Figure 8B

FASN/HepG2 and Huh7

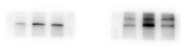

SIAH1/HepG2 and Huh7

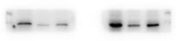

GAPDH/HepG2 and Huh7

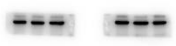

Figure 8C

SIAH1/HepG2 and Huh7

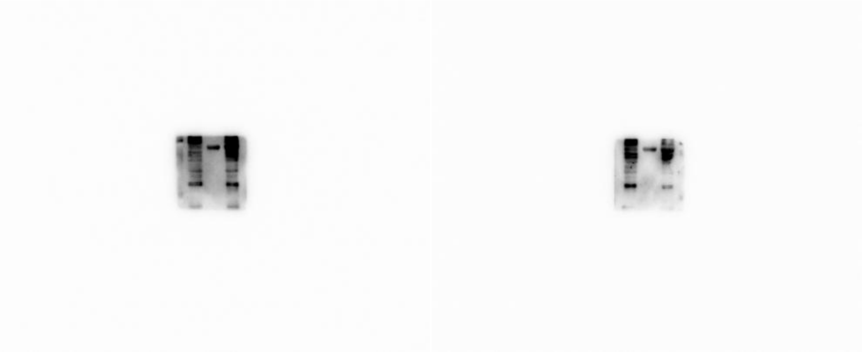

FASN/HepG2 and Huh7

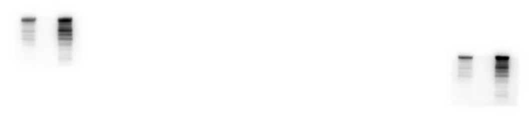

Figure 8D

FASN/HepG2 and Huh7

His-SIAH1/HepG2 and Huh7

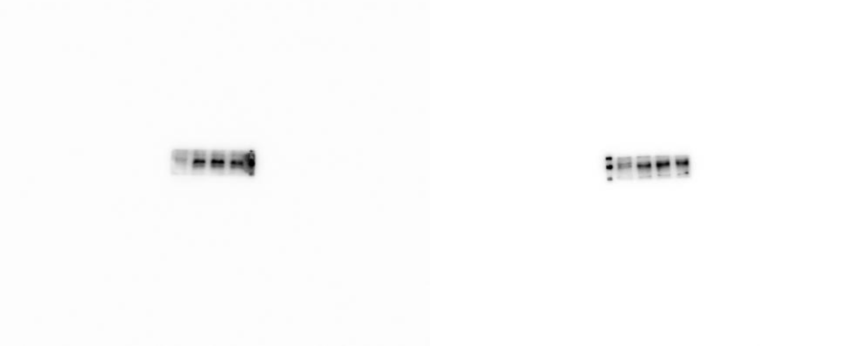

GAPDH/HepG2 and Huh7

Figure 8E

FASN/HepG2 and Huh7

 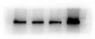

His-SIAH1/HepG2 and Huh7

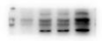 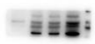

GAPDH/HepG2 and Huh7

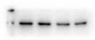 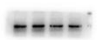

Figure 8F

FASN/HepG2 and Huh7

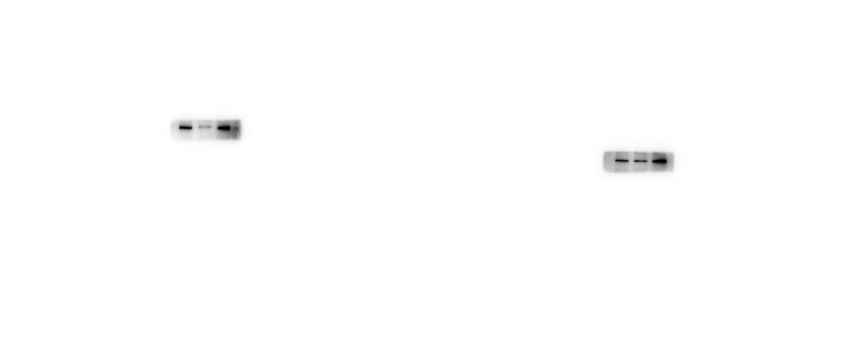

His-SIAH1/HepG2 and Huh7

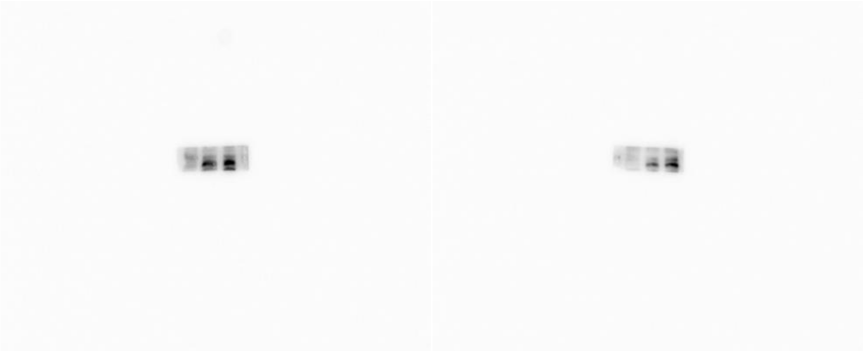

GAPDH/HepG2 and Huh7

Figure 8G

Ub/HepG2 and Huh7

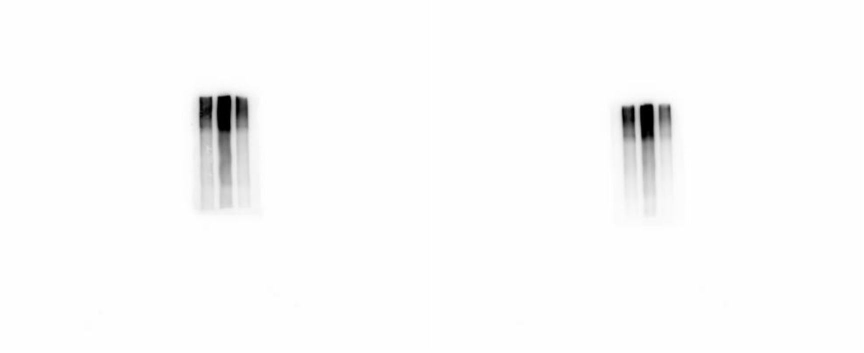

FASN/HepG2 and Huh7

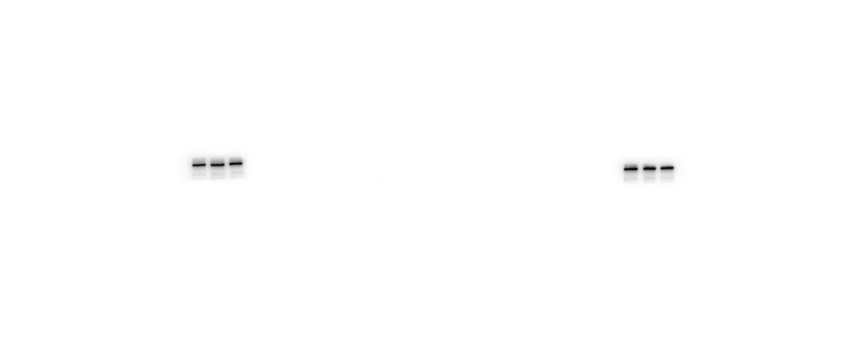

His-SIAH1/HepG2 and Huh7

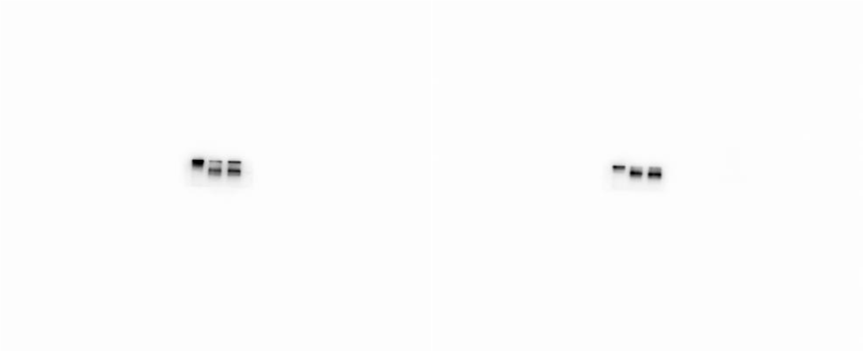

GAPDH/HepG2 and Huh7

Figure 8H

FASN

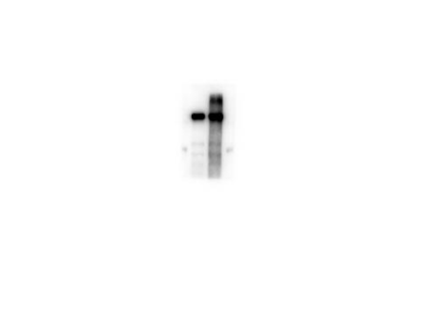

Figure 8I

HA/HepG2 and Huh7

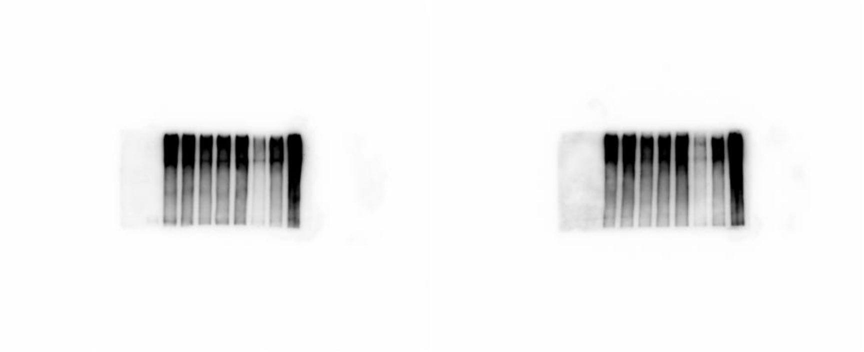

FASN/HepG2 and Huh7

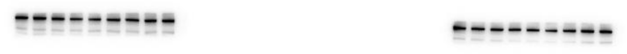

His-SIAH1/HepG2 and Huh7

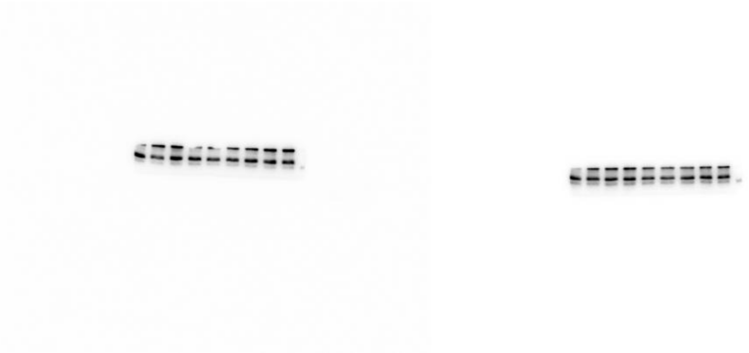

GAPDH/HepG2 and Huh7

Figure 8J

FASN/HepG2 and Huh7

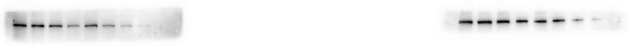

His-SIAH1/HepG2 and Huh7

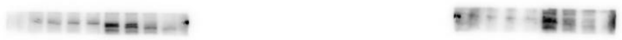

GAPDH/HepG2 and Huh7

Figure 9

Figure 9A

FSCN1/HepG2 and Huh7

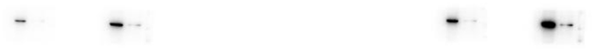

GAPDH/HepG2 and Huh7

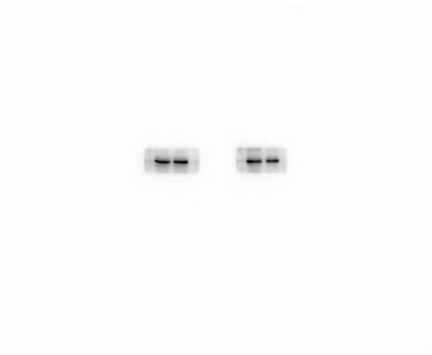

Figure 9B

FSCN1/HepG2 and Huh7

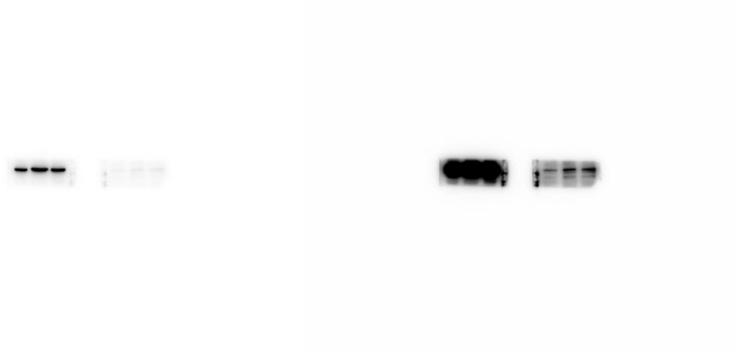

GAPDH/HepG2 and Huh7

Figure 9F

FSCN1/HepG2 and Huh7

3×Flag-FASN/HepG2 and Huh7

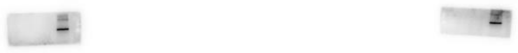

His-SIAH1/HepG2 and Huh7

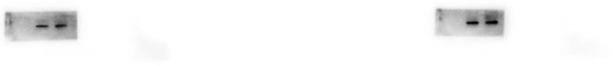

GAPDH/HepG2 and Huh7

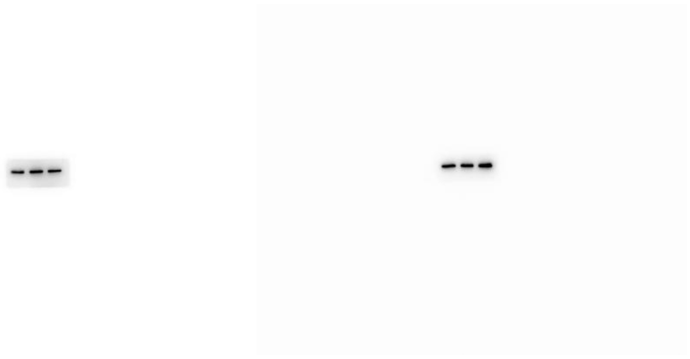

Figure 9L

FSCN1

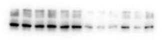

FASN

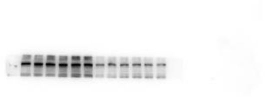

UCHL5

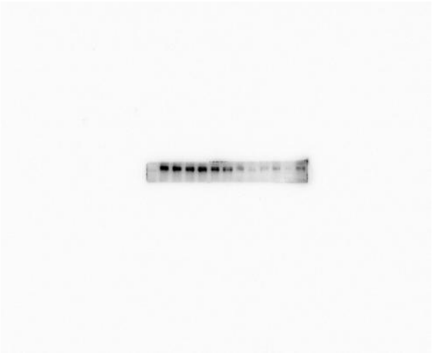

ADRM1

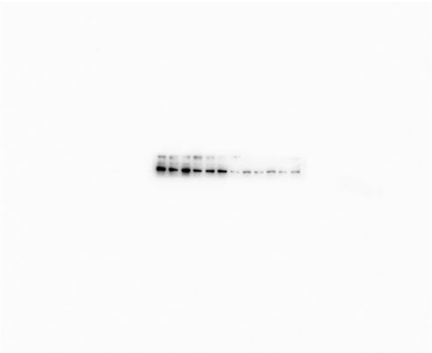

SIAH1

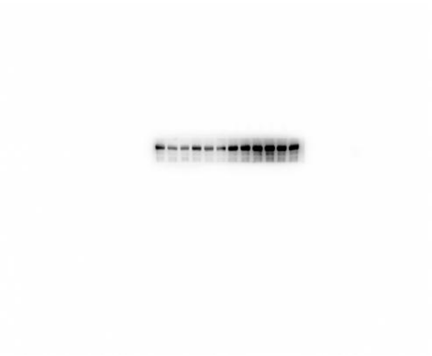

GAPDH

Supplementary Figure 2

Supplementary Figure 2A

ADRM1/HepG2 and Huh7

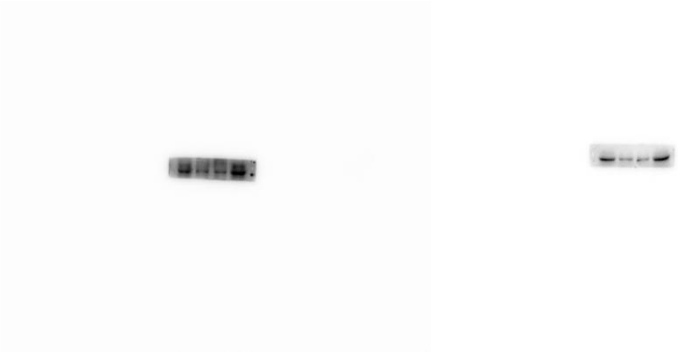

His-SIAH1/HepG2 and Huh7

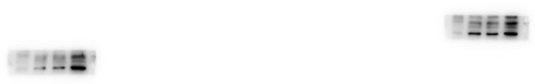

GAPDH/HepG2 and Huh7

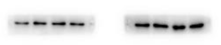

Supplementary Figure 2B

ADRM1/HepG2 and Huh7

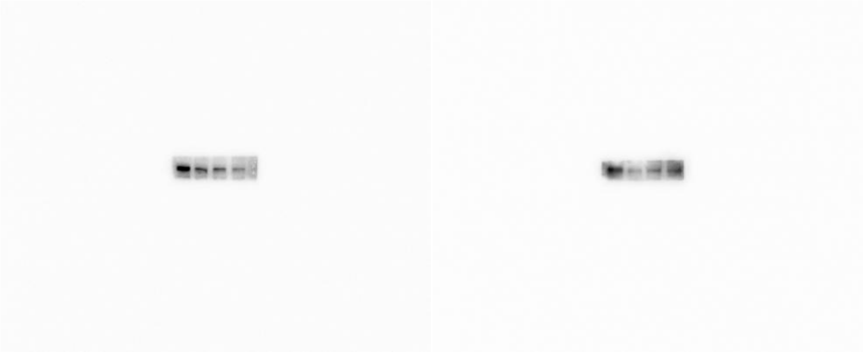

His-SIAH1/HepG2 and Huh7

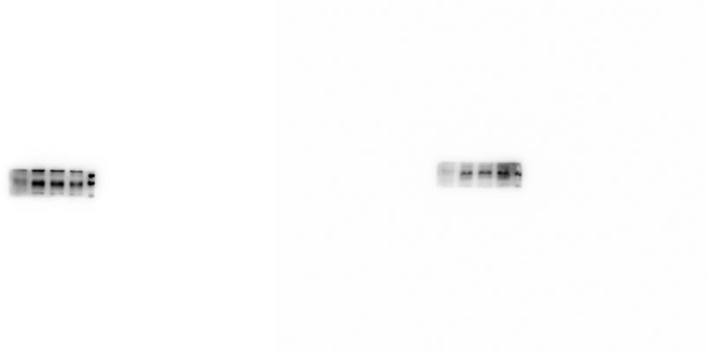

GAPDH/HepG2 and Huh7
